# Research on Variable Parameter Color Image Encryption Based on Five-Dimensional Tri-Valued Memristor Chaotic System

**DOI:** 10.3390/e26070536

**Published:** 2024-06-22

**Authors:** Pan Wang, Lina Ding

**Affiliations:** School of Electronics and Information Engineering, Heilongjiang University of Science and Technology, Harbin 150022, China

**Keywords:** voltage-controlled tri-valued memristor, different structures’ synchronous control, variable parameter

## Abstract

To construct a chaotic system with complex characteristics and to improve the security of image data, a five-dimensional tri-valued memristor chaotic system with high complexity is innovatively constructed. Firstly, a pressure-controlled tri-valued memristor on Liu’s pseudo-four-wing chaotic system is introduced. Through analytical methods, such as Lyapunov exponential map, bifurcation map and attractor phase diagram, it is demonstrated that the new system has rich dynamical behaviors with periodic limit rings varying with the coupling parameter of the system, variable airfoil phenomenon as well as transient chaotic phenomenon of chaos-periodic depending on the system parameter and chaos-quasi-periodic depending on the memristor parameter. The system is simulated with dynamic circuits based on Simulink. Secondly, the differently structured synchronous controls of chaotic systems are realized using a nonlinear feedback control method. Finally, based on the newly constructed five-dimensional chaotic system, a variable parameter color image encryption scheme is proposed to iteratively generate varying chaotic pseudo-random sequences by varying the system parameters, which will be used for repetition-free disambiguation, additive modulo left-shift diffusion and DNA encryption for the three components of RGB of the color image after chunking. The simulation results are analyzed by histogram, information entropy, adjacent pixel correlation, etc., and the images are tested using differential attack, noise attack and geometric attack, as well as analyzing the PSNR and SSIM of the decrypted image quality. The results show that the encryption method has a certain degree of security and can be applied to medical, military and financial fields with more complex environmental requirements.

## 1. Introduction

With the continuous development of information technology, ensuring information security has become an important issue, and images, as the most frequently used multimedia tools, are widely used in medical, military, education, finance and other fields, often facing the risk of leakage, resulting in incalculable losses. Images are characterized by large amounts of data, redundancy and high correlation, so traditional encryption algorithms are not suitable for image encryption [1], and it is crucial to design an effective encryption algorithm to protect the relevant information of images. Chaotic systems are suitable for image encryption due to their properties such as ergodicity, initial value sensitivity and non-periodicity, which are similar to cryptography. The common chaotic systems are logistic mapping, Henon mapping, Lorenz chaotic system, etc. The research history of chaos theory can be traced back to the late 19th century at the earliest, but it is only in recent decades that it has begun to be applied to image encryption. Previous studies have shown that applying chaos to encryption algorithms can effectively improve the security of encryption algorithms.

In recent years, an in-depth study of the electrical characteristics of memristors and their application to chaotic systems has become one of the important research directions, and most of the studies on memristor chaotic systems are centered around magnetically controlled memristors and charge-controlled memristors. In 2024, Li Ping et al. proposed an image encryption algorithm combining the disruption–diffusion algorithm by constructing a five-dimensional memristor chaotic system using a magnetically controlled memristor [2]. In 2023, Liu Ni constructed a four-dimensional fractional-order chaotic system using magnetically controlled memristors, designed a diffusion algorithm encryption scheme based on heteroscedasticity and applied it to a power system, and the experiment proved that the encryption scheme has good security performance [3]. In 2023, Du Chuanhong designed a conservative system using a charge-controlled memristor to make a pseudo-random signal generator and used it for image encryption work [4]. In summary, the sequences generated by the chaotic system based on magnetically controlled memristors and charge-controlled memristors have strong pseudo-randomness and can be well applied in image encryption.

A number of scholars have begun to study other types of memristors. In 2020, Wang Xiaoyuan et al. first proposed the concept and mathematical model of a multi-valued memristor, deeply analyzed the mathematical model of a tri-valued memristor [5] and constructed a new four-dimensional tri-valued memristor chaotic system in the same year, which was proved experimentally to have a complex dynamical behavior [6]. With the continuous efforts of the team, in 2023, two four-dimensional memristor chaotic systems were constructed by introducing two-valued and tri-valued memristors into Chen’s system, respectively, and the differences between the two chaotic systems were compared from several angles, proving that the chaotic system constructed by tri-valued memristors could produce more complex dynamics [7]. In summary, a tri-valued memristor not only enriches the types of nonlinear systems compared to a two-valued and continuous memristor, but also broadens the design of chaotic systems, increases the stability and robustness of the system and produces rich and diverse dynamic behaviors. At this stage, the research on tri-valued memristor chaos is relatively small and limited to the basis of four-dimensional chaotic systems, and the constructed four-dimensional chaotic systems have the problems of simple structure and low sequence randomness, whereas the high-dimensional chaotic systems have more complex dynamical behaviors and a richer class of stochastic phenomena in comparison with the four-dimensional chaotic systems [8,9,10,11]. Therefore, it is challenging research to introduce tri-valued memristors into high-dimensional chaotic systems, and in this paper, we address the above problems by constructing high-dimensional chaotic systems using tri-valued memristors to produce richer dynamical behaviors and more stochastic sequences.

Synchronous control is the key to achieving confidential communication and has important applications in the fields of communication, secure transmission and information processing [12,13,14]. In 2019, Yu Fei et al. achieved synchronization between five-dimensional memristor hyperchaotic systems with different structures by actively controlling the synchronization and successfully applied it in the field of confidential communication [15]. In 2021, Liu Li combined a newly constructed five-dimensional memristor hyperchaotic system with a chaotic system of the same number of dimensions to achieve different structures’ synchronous control of the two systems [16]. In 2023, Dong Wu et al. designed a combinatorial synchronization scheme for differently structured hyperchaotic systems based on finite time theory using the backstepping method to improve the security of the secure communication system and verified the effectiveness of the scheme through experimental simulation [17]. In summary, the synchronous control of chaotic systems lays the foundation for the development of the communication field. Although some progress has been made in the research on the synchronous control of differently structured chaotic systems, the theoretical research on the synchronization of the high-dimensional differently structured chaotic systems containing different memristors is still insufficient. In this paper, we design a new synchronous controller to achieve the synchronous control of the newly constructed tri-valued memristor chaotic system and the magnetically controlled memristor chaotic system of the five-dimensional differently structured chaotic system synchronization control.

Chaotic systems have the properties of initial value sensitivity, boundedness, ergodicity and randomness [18], which can produce rich dynamical behaviors and have important applications in the fields of image encryption, neural networks and confidential communications. Chaotic encryption has the advantages of larger key space and faster encryption speed compared to traditional image encryption methods, which has become one of the important topics in the field of nonlinear research [19,20,21]. In 2022, Wang Simiao et al. proposed a chaotic encryption scheme combining three-dimensional logical mapping and Secure Hash Algorithm-3 to improve encryption effectiveness [22]. In 2023, Li Jian et al. proposed a semiconductor 32 image encryption method based on a smooth memristor with a conservative memristor chaotic system, which provided a reference for further research on the application of conservative memristor chaotic systems in the field of image encryption [23]. In 2023, Wang Yiming et al. designed a fast image encryption algorithm with an adaptive mechanism based on a 3D chaotic system [24]. The above research is limited to the encryption of a chaotic system with fixed parameters, the key space is insufficient, the complexity is low, it is easy to be affected by the short period and chaotic degradation and the security is poor as the same key flow is used for each image. Therefore, it is necessary to improve the current encryption method of the chaotic image to enhance its security and reliability. In this paper, we will focus on the above problems, and based on the principle of one-key-at-a-time, we will design a variable parameter encryption scheme, which can solve the above problems well.

Based on the above research, this paper further enriches the type of chaotic system based on a tri-valued memristor and proposes a more effective secrecy scheme based on this system. The remainder of this study is structured as follows. Section 2 constructs a five-dimensional tri-valued memristor system, which is verified as a chaotic system without equilibrium using a combination of theory and experiments. Section 3 explores the effects of the system parameters and the memristor parameters on the dynamical properties of the system and demonstrates that the system parameters are highly sensitive and can be better applied in image encryption. Section 4 uses the Simulink circuit implementation to simulate the chaotic system and verifies the correctness of the chaotic system. Section 5 uses a nonlinear feedback synchronization method to design a nonlinear feedback controller to synchronize a chaotic system containing two different memristors, which lays the foundation for the field of confidential communication applications. Section 6 summarizes the work of this paper and provides a future outlook.

## 2. Construction of a Five-Dimensional Tri-Valued Memristor Chaotic System

### 2.1. Voltage-Controlled Tri-Valued Memristor Model

**Figure 1:**
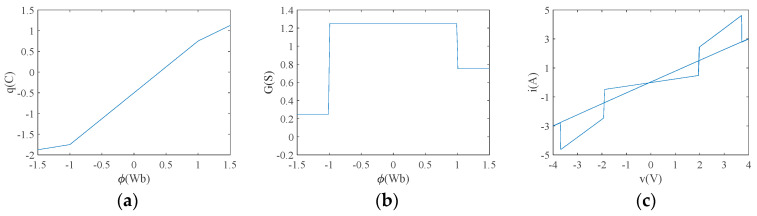
Characteristic curves of tri-valued memristor: (**a**) φ−q curve; (**b**) φ−G curve; (**c**) v-i characteristic curve.

In 2020, Wang Xiaoyuan et al. [5] proposed a voltage-controlled tri-valued memristor, which describes the adopted tri-valued memristor model φ−q relation in terms of an asymmetric segmented linear function, as shown in Equation (1):

(1) $$q=e_{0}+a_{0}\varphi +b_{0}\left|\varphi +c_{0}\right|-d_{0}\left|\varphi -c_{0}\right|$$

where the parameters $a_{0}$, $b_{0}$, $c_{0}$, $d_{0}$ and $e_{0}$ are non-zero constants and $c_{0}$ is positive (Equation (1)). Both sides of the equation are simultaneously derived for the flux φ to obtain the φ−G relation for this tri-valued memristor model as a segmented functional relation, as shown in Equation (2):

(2) $$\begin{array}{ll} \frac{\mathrm{dq}}{d\varphi } & =G(\varphi )=a_{0}+b_{0}\mathrm{sgn}(\varphi +c_{0})-d_{0}\mathrm{sgn}(\varphi -c_{0})\\ & =\left\{\begin{array}{l} a_{0}-b_{0}+d_{0},\varphi <-c_{0}\\ a_{0}+b_{0}+d_{0},-c_{0}<\varphi <c_{0}\\ a_{0}+b_{0}-d_{0},\varphi >c_{0} \end{array}\right. \end{array}$$

where G(φ) is the memristor conductance of the memristor in Siemens (S), and sgn denotes the sign function. Let $a_{0}=0.5$, $b_{0}=0.5$, $c_{0}=1$, $d_{0}=0.25$ and $e_{0}=0.75$, and the corresponding curves φ−q and φ−G are shown in Figure 1a,b. From Figure 1, the memristor is modeled as a three-part line segment passing through the origin, exhibiting three stable memristor values of 0.25 S, 1.25 S and 0.75 S. Figure 1b shows more intuitively the effect of the change in magnetic flux on the tri-valued memristor value of the voltage-controlled tri-valued memristor. When φ<−1, G(φ)=0.25 S; when −1<φ<1, G(φ)=1.25 S; and when φ>1, G(φ)=0.75 S. Applying an excitation voltage with a sinusoidal signal of $v=v_{0}\sin(2\pi ft)$ to this tri-valued memristor model, taking $v_{0}=4$ V, f=0.159 Hz and an initial value of φ(0)=−1.5, the v-i characteristic curve can be obtained, as shown in Figure 1c. The v-i plane shows a tight “8” contraction hysteresis curve over the origin, which satisfies the non-volatile nature of the memristor and proves that the voltage-controlled tri-valued memristor is effective.

### 2.2. Construction of the System

Liu’s pseudo-quadruple chaotic system [25] to be improved is shown in Equation (3).

(3) $$\left\{\begin{array}{l} \overset{•}{x}=\mathrm{ax}-\mathrm{byz}\\ \overset{•}{y}=-cy+xz\\ \overset{•}{z}=-dz+xy \end{array}\right.$$

In Equation (3), x, y and z are the state variables of the system; a, b, c and d are the system parameters. On this basis, a tri-valued memristor is added to the second equation to form a state feedback, a state variable w is added to the third equation, the sum of state variables x and y are used as the fourth equation and the sum of state variables w and u are used as the fifth equation, thus obtaining a new five-dimensional tri-valued memristor chaotic system with the following mathematical model:

(4) $$\left\{\begin{array}{l} \overset{•}{x}=\mathrm{ax}-\mathrm{byz}\\ \overset{•}{y}=-cy+xz-G(u)\\ \overset{•}{z}=-dz+xy-w\\ \overset{•}{w}=x+y\\ \overset{•}{u}=w+u \end{array}\right.$$

In Equation (4), x, y, z, w and u are the state variables of the system; a, b, c and d are the system parameters.

### 2.3. Validation of the System

Simulation software MATLAB 2018a is used to verify whether the newly constructed five-dimensional memristor system is chaotic, and the system is analyzed by the 0–1 test, time-domain waveform map and attractor phase diagram, as follows:

The 0–1 test [8], which determines whether the sequences generated by the system exhibit chaotic behavior, shows an interesting feature: it provides a simple and intuitive test for the trajectory of the system in the (p,s) plane, regardless of whether the dynamical system is chaotic. Let the discrete time series φ(i) at sampling time j=1, 2, …, N, with c chosen as a random constant in the interval (0,2π), define the functions p(n) and q(n) as follows:

(5) $$p(n)=\sum_{j=1}^{n}\varphi (j)\cos(\theta (j)),n=1,\;2,\;\ldots ,\;N$$

(6) $$s(n)=\sum_{j=1}^{n}\varphi (j)\sin(\theta (j)),n=1,\;2,\;\ldots ,\;N$$

where φ(j) is the observable data set and parameter $\theta (j)=jc+\sum_{i=1}^{j}\varphi (i)$, j=1, 2, …, N.

Based on the functions p(n) and q(n), the mean square displacement M(n) is as follows:

(7) $$M(n)=M_{c}(n)-{\left(E(\varphi )\right)}^{2}(1-\cos nc)/(1-\cos c)$$

Included among these,

(8) $$M_{c}(n)=\underset{N\to \infty }{\lim}\frac{1}{N}\sum_{J=1}^{N}\left[(p(j+n)-{\left(p(j)\right)}^{2}-(q(j+n)-{\left(q(j)\right)}^{2}\right]$$

(9) $$E(\varphi )=\underset{N\to \infty }{\lim}\frac{1}{N}\sum_{j=1}^{N}\varphi (j)$$

If the trajectories of the functions p(n) and q(n) appear to be in irregular Brownian motion, the system is in a chaotic state; if the trajectories of p(n) and q(n) are bounded, the system is in a periodic state.

The asymptotic growth rate $K_{c}$ of the mean square displacement M(n) is defined to be

(10) $$K_{c}=\underset{N\to \infty }{\lim}\log M(n)/\log n$$

The test method can determine the chaotic properties of a time series of finite length simply based on whether the asymptotic growth rate $K_{c}$ is closer to 0 or 1, as shown in Table 1.

**Figure 2:**
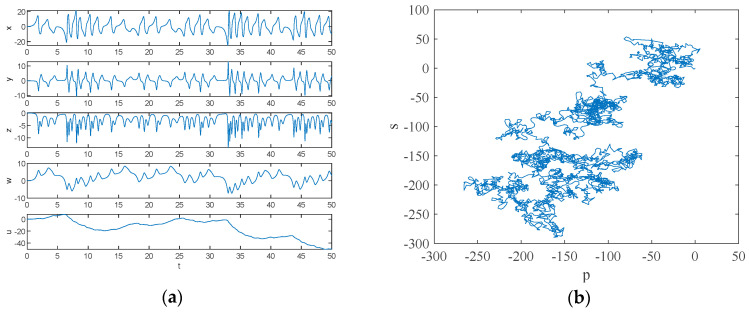
Time-domain waveform and 0–1 test plot with parameters a=3, b=3, c=8 and d=5: (**a**) time-domain waveform; (**b**) 0–1 test plot.

**Figure 3:**
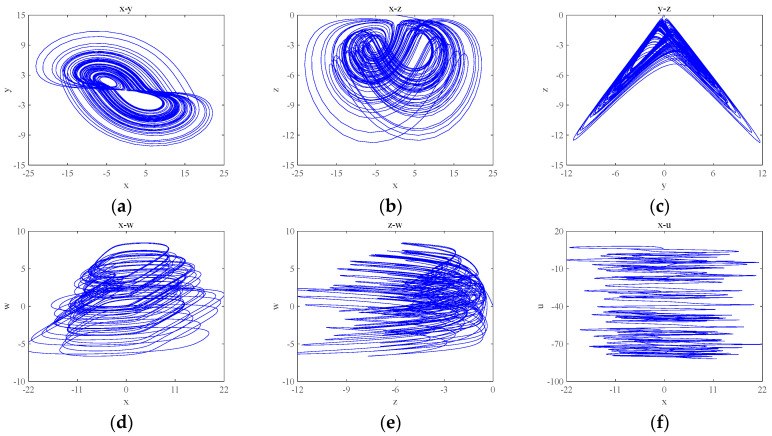
Basic phase diagrams of the system: (**a**) the x−y plane; (**b**) the x−z plane; (**c**) the y−z plane; (**d**) the x−w plane; (**e**) the z−w plane; and (**f**) the x−u plane.

According to the 0–1 test method, the dynamic system will provide a simple visual test in the plane of (p,s). The trajectory within (p,s) provides a judgement on the state that the dynamic system is in. Let a=3, b=3, c=8 and d=5, and the initial condition is (0.1, 0.1, 0.1, 0.1, 0.1). Numerical simulation using MATLAB 2018a yields the time-domain waveform and 0–1 test plot of the system, which are shown in Figure 2, and the attractor phase diagrams of the x−y, x−z, y−z, x−w, z−w and x−u planes obtained by numerical simulation using MATLAB 2018a are shown in Figure 3. As can be seen from Figure 2, the 0–1 test plot is an irregular Brownian motion, and the value of $K_{c}$ is 1. The time-domain waveform trajectory of the system is always an irregular clutter wave.

In summary, the time-domain waveform map of the system is non-periodic and the 0–1 test map shows Brownian motion, so the newly constructed system is chaotic.

### 2.4. Equilibrium Stability and Dissipative Analysis

Let the right-hand side of the system (4) be zero, and the condition that the system has an equilibrium point can be obtained as x=0, y=0, z=0, w=0, u=0 and G(u)=0. From the defined tri-valued memristor model, it can be seen that the tri-valued memristor exhibits only three stable memristor conductance values of 0.25 S, 1.25 S and 0.75 S. The absence of G(u)≠0 indicates that the above condition for the existence of equilibrium points does not hold and, therefore, the chaotic system has no equilibrium points and the attractors produced are all hidden attractors.

In order to obtain the dissipation of the system, Equation (11) is used for calculation:

(11) $$\nabla v=\frac{\dot{\partial x}}{\partial x}+\frac{\dot{\partial y}}{\partial y}+\frac{\dot{\partial z}}{\partial z}+\frac{\dot{\partial w}}{\partial w}+\frac{\dot{\partial u}}{\partial u}=a-c-d+1$$

When a=3, b=3, c=8 and d=5, the initial condition is (0.1, 0.1, 0.1, 0.1, 0.1), respectively, ∇v=−9 is calculated, which indicates that the system is dissipative, the volume of the phase space will be contracted exponentially to $e^{-9t}$ and all the trajectories of the system are compressed to 0, which is one of the most important bases for the existence of chaotic phenomena in the system.

## 3. Analysis of the Dynamical Behavior of the System

Chaotic properties are to some extent susceptible to the influence of system parameters, i.e., small perturbations in the system parameters are prone to cause changes in the dynamical behavior of the system, and this sensitivity can limit the application of chaotic systems in practice, so it is crucial to explore the influence of system parameters on the dynamics of chaotic systems [26]. The Lyapunov exponential and bifurcation diagrams are classical methods for analyzing the behavior of nonlinear dynamics.

The Lyapunov exponent is a numerical feature of the average exponential divergence rate of neighboring trajectories in phase space and is one of several numerical features used to identify chaotic motion. Whether a particular system is chaotic or not can be visualized by the image of the Lyapunov exponential spectrum, where only one positive Lyapunov exponent implies chaos, and two or more positive Lyapunov exponents imply hyperchaotic and the trajectory will exhibit greater complexity and unpredictability. The equation for the Lyapunov exponent [27] is as follows:

(12) $$\lambda_{Li}=\underset{t\to \infty }{\lim}\frac{1}{T}\ln\left|\frac{dy(T)}{{\mathrm{dy}}_{0}}\right|$$

where $\lambda_{Li}$ is the i Lyapunov index, y(T) is the state vector at time T and $y_{0}$ is the initial state vector.

The bifurcation diagram of the chaotic system is a tool for visualizing stability and periodicity in the evolution of a system, usually using the system parameters as horizontal coordinates and the values of the system state, stability points or periodic orbits as vertical coordinates. In chaotic systems, small parameter changes may lead to significant changes in the behavior of the system, resulting in bifurcation. The different attractor states of the system can be observed from it, and if the bifurcation diagram appears as one or more straight lines consisting of dots, it indicates that the system is in a periodic or multiply-periodic state. If the bifurcation diagram appears with patches of dots, it indicates that the system is in a chaotic state.

Therefore, by fixing the other parameters, the data variations in Lyapunov exponent spectrum and bifurcation diagram in different ranges of the system coupling parameters a, d and memristor parameter $b_{0}$, respectively, are observed to understand the complex dynamic properties of the system.

### 3.1. Dependent on the Dynamics of Parameter a

**Figure 4:**
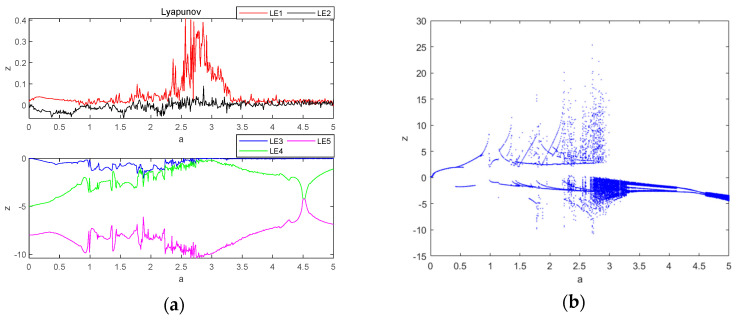
Lyapunov exponent spectrum and bifurcation diagram of chaotic system with $a\in \left[0,5\right]$: (**a**) Lyapunov exponent spectrum; (**b**) bifurcation diagram.

Let parameter a be a variable control parameter, when b=3, c=8 and d=5, the initial condition is (0.1, 0.1, 0.1, 0.1, 0.1), the fixed step time is 0.01 s and the total duration is t=200 s. The fourth-order Runge–Kutta method and wolf algorithm are used to simulate the Lyapunov exponent spectrum and bifurcation diagrams for the evolution of the system variable z within the parameter $a\in \left[0,5\right]$. The size of the value obtained from different sampling points when experimenting is not the same, which also depends on the performance of the computer, in the case of ensuring that the computer can run, as many as possible to obtain the sampling points, to improve the accuracy of the value, in which the Lyapunov exponential spectrum of 500 sampling points, bifurcation diagram sampling points of 500, the obtained image is shown in Figure 4. The principle adhered to is to take the second Lyapunov exponent value close to 0 as 0, and the value with a large difference from 0 is taken by the normal value, using this way of taking the value, which will result in a certain degree of error, so the attractor phase diagrams and the time-domain waveform diagrams are re-simulated at the special point and the critical point, to re-verify the data correctness as well as the correctness of the system state in the interval and the accuracy of the data as well as the state of the system are greatly improved correctness of the data and the system state. It can be seen that the system goes through three different states, first changing from periodic to chaotic and then to quasi-periodic: when $a\in \left[0,2.66\right]$, the system is in a periodic state; when $a\in \left[2.66,3.3\right]$, the system is in a chaotic state; and when $a\in \left[3.3,5\right]$, the system is in a quasi-periodic state. 

From Figure 4, the values of Lyapunov exponents of the system are $LE_{1}=0.1957$, $LE_{2}=0.002379$, $LE_{3}=-0.02751$, $LE_{4}=-0.2597$ and $LE_{5}=-9.91$. Based on the above values of Lyapunov exponents, the following can be obtained:

(13) $$\sum_{i=1}^{3}LE_{i}=0.170569>0$$

(14) $$\sum_{i=1}^{4}LE_{i}=-0.089131<0$$

The fractional dimension of the system is calculated using the wolf algorithm [28], as shown in Equation (15):

(15) $$D_{L}=j+\frac{1}{\left|L\left.E_{j+1}\right|\right.}\sum_{i+1}^{j}LE_{i}$$

where j is to ensure that $\sum_{i=1}^{j}LE_{i}\geq 0$ and $\sum_{i=1}^{j+1}LE_{i}\leq 0$, and thus one obtains the following:

(16) $$D_{L}=3+\frac{1}{\left|L\left.E_{4}\right|\right.}\sum_{1=1}^{3}LE_{3}=3+\frac{0.170569}{\left|-0.2597\right|}=3.657$$

It shows that the Lyapunov exponential dimension $D_{L}$ of the system is fractional dimensional, further verifying that the system is chaotic at this point.

**Figure 5:**
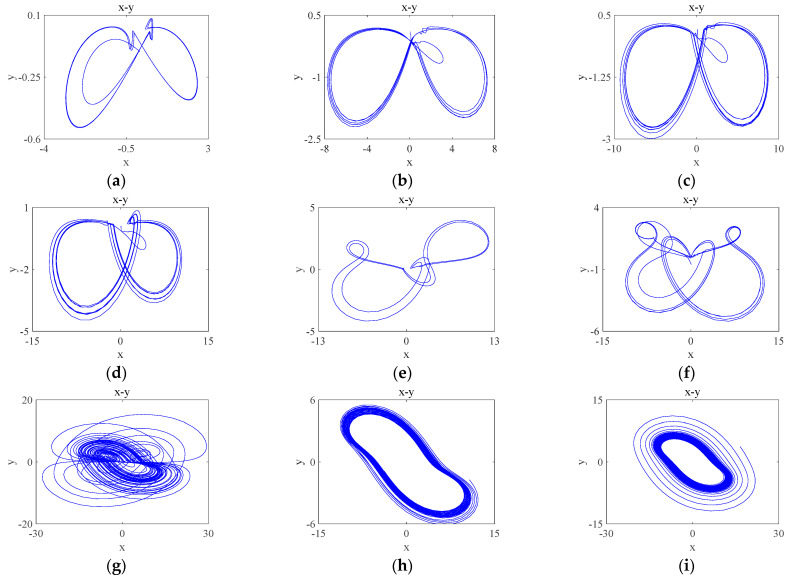
Phase portraits of the system with different parameter a (Table 2) on x-y plane: (**a**) a=0.3; (**b**) a=0.7; (**c**) a=0.8; (**d**) a=0.9; (**e**) a=1.2; (**f**) a=1.6; (**g**) a=2.8; (**h**) a=4.2; (**i**) a=4.8.

To investigate the periodic limit ring of the system, a number of parameter a values are selected, as shown in Table 2, and their attractor phase diagrams are shown in Figure 5, where Table 2 corresponds to the attractor phase diagrams of Figure 5. It can be seen that the system has a periodic limit ring and chaotic attractor dependent on the different parameters a.

### 3.2. Dependent on the Dynamics of Parameter d

**Figure 6:**
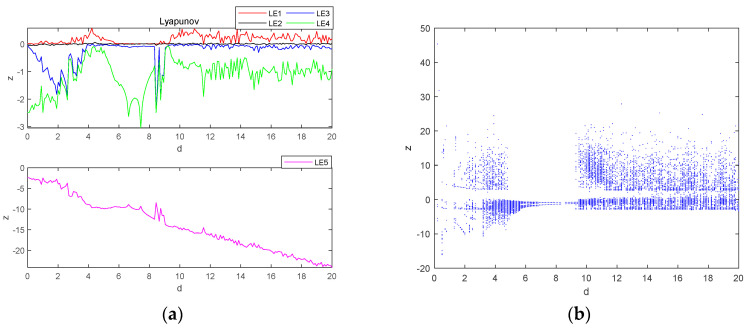
Lyapunov exponent spectrum and bifurcation diagram of chaotic system with $d\in \left[0,20\right]$: (**a**) Lyapunov exponent spectrum; (**b**) bifurcation diagram.

Let parameter d be a variable control parameter, when a=3, b=3 and c=8, the initial condition is (0.1, 0.1, 0.1, 0.1, 0.1), the fixed step time is 0.01 s and the total duration is t=200 s. The fourth-order Runge–Kutta method and wolf algorithm are used to simulate the Lyapunov exponent spectrum and bifurcation diagrams for the evolution of the system variable z within the parameter $d\in \left[0,30\right]$. The Lyapunov exponent spectrum is sampled at 600 points and the bifurcation diagram at 600 points, and the obtained images are shown in Figure 6. 

As can be seen in Figure 6, when parameter d is used as a variable control parameter, the system varies between three states: periodic, chaotic and quasi-periodic. In system $d\in \left[0,3.2\right]$ it is in a periodic state; in systems $d\in \left[3.2,5.5\right]$ and $d\in \left[9.2,30\right]$, it is in a chaotic state; and in system $d\in \left[5.5,9.2\right]$, it is in a quasi-periodic state.

**Figure 7:**
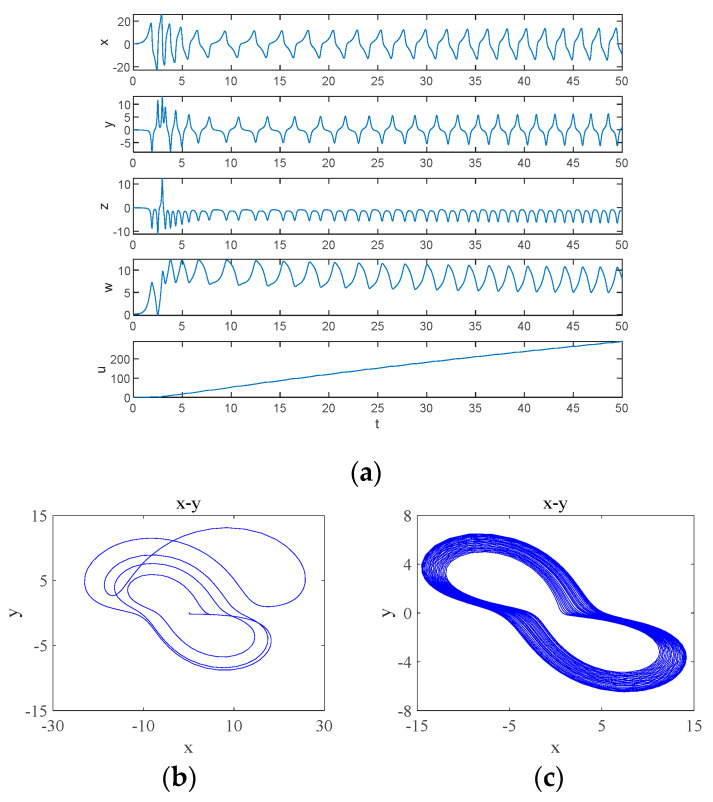
Transient chaos phenomenon of the system from chaos-period when d=9: (**a**) time-domain waveform; (**b**) transient chaos at $t\in \left[0,20\right]$; (**c**) steady state period at $t\in \left[20,50\right]$.

When d=9, the system undergoes a transition from a quasi-periodic state to a chaotic state, and the simulation obtains the time-domain waveform of the system as shown in Figure 7a, where the system behaves chaotically for a shorter period and enters into a periodic behavior after a certain period, which is referred to as transient chaos, which is due to the presence of non-attractive saddle points in the phase space [29]. At $t\in \left[0,20\right]$, the system is in a chaotic state and its attractor phase diagram is shown in Figure 7b, and at $t\in \left[20,50\right]$, the system is in a periodic state and its attractor phase diagram is shown in Figure 7c.

**Figure 8:**
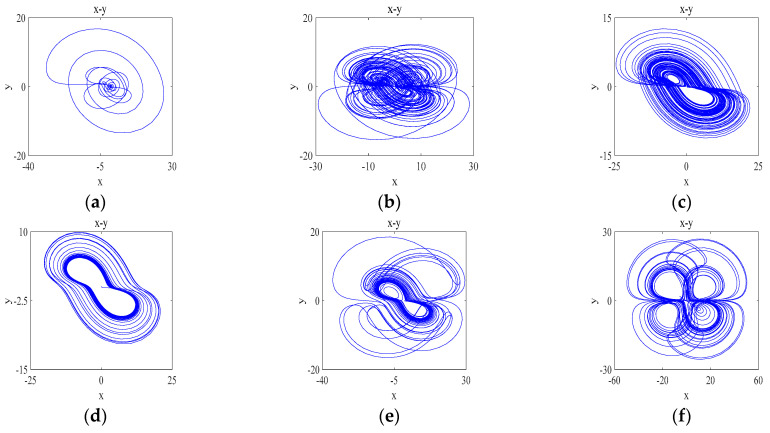
Attractor phase diagrams for variable wings dependent on parameter d: (**a**) d=1; (**b**) d=4; (**c**) d=5; (**d**) d=8; (**e**) d=10; (**f**) d=30.

The different types of attractors that the system will produce when the parameter d is varied: when d=1 the periodic state attractor of the system is shown in Figure 8a; when $d\in \left[3.2,4.7\right]$ the system has two positive values of the Lyapunov exponent and the system has significant quadruple-winged hyperchaos in the phase plane, such that d=4, whose attractor phase diagram is shown in Figure 8b; when $d\in \left[4.7,5.5\right]$ the system has significant two-wing hyperchaos in the phase plane, let d=5 and its attractor phase diagram is shown in Figure 8c; when d=8 the quasi-periodic state attractor of the system is shown in Figure 8d; when d=10 the system is in a weakly chaotic state and its attractor phase diagram is shown in Figure 8e; and when $d\in \left[9.2,30\right]$, the system is in a chaotic state and its attractor still presents a quadruple-winged state, but its quadruple-winged attractor is more regular compared to $d\in \left[3.2,4.7\right]$. Let d=30 and its attractor phase diagram is shown in Figure 8f. Thus, the system has a large adjustable space for the parameter d and has a variable wing phenomenon dependent on the parameter d.

### 3.3. Dependence on the Kinetic Properties of the Memristor Parameter $b_{0}$

**Figure 9:**
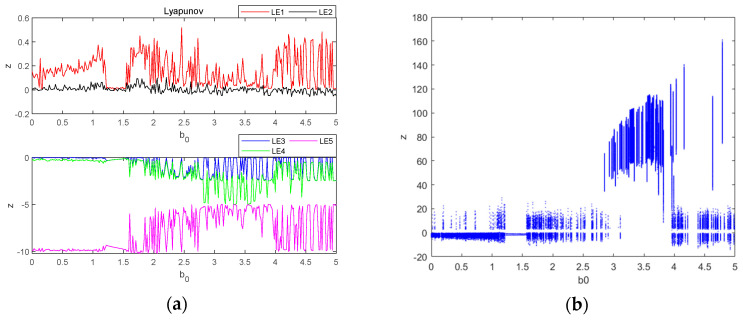
Lyapunov exponent spectrum and bifurcation diagram of chaotic system with $b_{0}\in \left[0,5\right]$: (**a**) Lyapunov exponent spectrum; (**b**) bifurcation diagram.

Let parameter $b_{0}$ be a variable control parameter, when a=3, b=3, c=8 and d=5, the initial condition is (0.1, 0.1, 0.1, 0.1, 0.1), the fixed step time is 0.01 s and the total duration is t=200 s. The fourth-order Runge–Kutta method and wolf algorithm are used to simulate the Lyapunov exponent spectrum and bifurcation diagrams for the evolution of the system variable z within the parameter $b_{0}\in \left[0,5\right]$. The Lyapunov exponent spectrum is sampled at 500 points and the bifurcation diagram at 500 points, and the obtained images are shown in Figure 9. 

From Figure 9, it can be seen that when parameter $b_{0}$ is used as a variable control parameter, the system is in the three states of periodic, chaotic and quasi-periodic and constantly switches between the two states of periodic and chaotic, as shown in Table 3.

**Figure 10:**
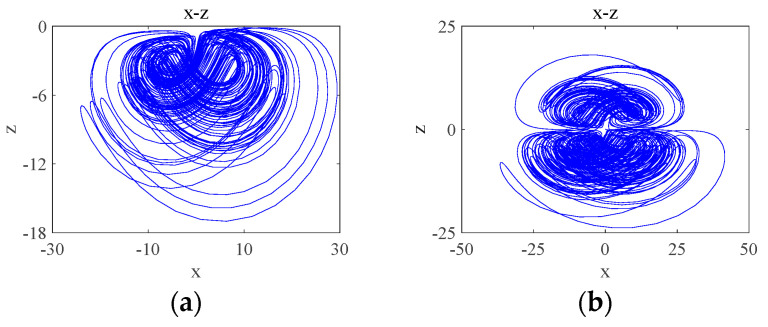
Attractor phase diagrams for variable wings dependent on parameter $b_{0}$: (**a**) $b_{0}=1$; (**b**) $b_{0}=4$.

The different types of attractors that the system will produce when the parameter $b_{0}$ is varied are as follows: when $b_{0}=1$, the attractors of the system present a chaotic state, as shown in Figure 10a, at which time the system (4) has two positive Lyapunov exponent values and the system has significant biplane hyperchaos in the phase plane; when $b_{0}=4$, the phase diagram of the attractor obtained from the simulation is shown in Figure 10b and the chaotic attractor presents a four-winged state. Thus, the memristor parameter $b_{0}$ of the system likewise has a large adjustable space, and the system has a variable wing phenomenon that depends on the memristor parameter $b_{0}$.

**Figure 11:**
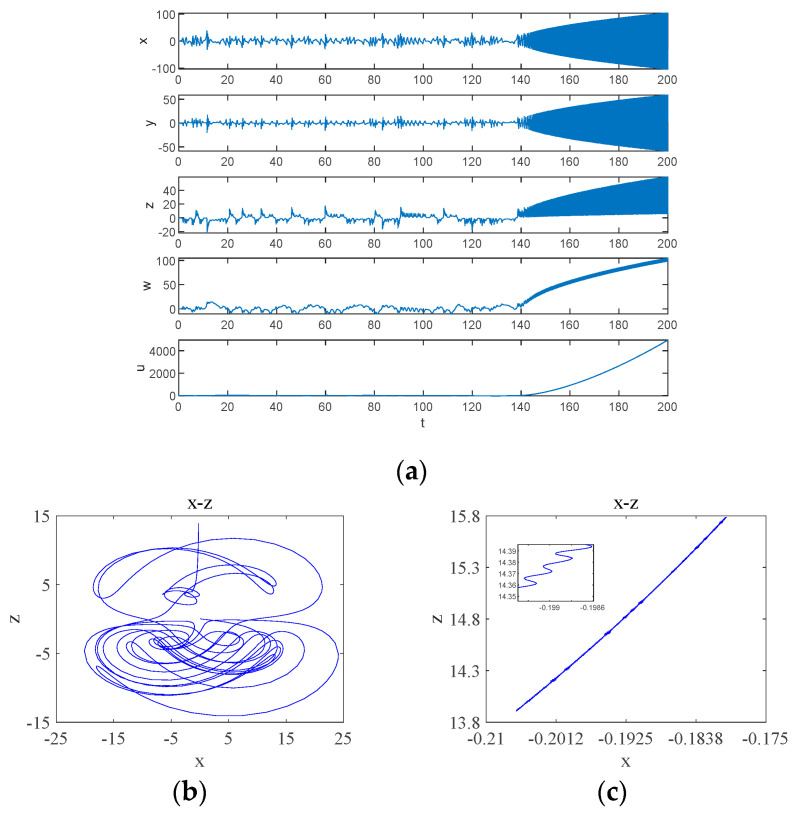
Time-domain waveform and attractor phase diagrams: (**a**) time-domain waveforms; (**b**) transient chaos at $t\in \left[0,140\right]$; (**c**) steady state period at $t\in \left[140,200\right]$.

When parameter $b_{0}$ is in the quasi-periodic state interval, the system will have transient chaos phenomenon, which is different from the transient chaos phenomenon depending on the coupling parameter d in Figure 7, and this time, the transient chaos phenomenon is a transition from chaotic to quasi-periodic state. $b_{0}=3.5$ is taken randomly in the quasi-periodic $b_{0}\in \left[2.81,3.94\right]$ interval, and the simulation yields the time-domain waveform as shown in Figure 11a. The system exhibits chaotic behavior on a finite time scale, transforming to a quasi-periodic state after a period of time: inside $t\in \left[0,140\right]$, the system is in a chaotic state and its attractor phase diagram is shown in Figure 11b; inside $t\in \left[140,200\right]$, the system is in a quasi-periodic state and its attractor phase diagram is shown in Figure 11c.

### 3.4. Complexity Analysis

The spectral entropy value of a chaotic system (SE) [30] is an important index for evaluating the complexity and stochasticity of chaotic sequences, which as an optimization algorithm of approximate entropy approximation can measure the complexity of chaotic sequences and can reflect the structural complexity of chaotic systems. In the field of communication, the system complexity is closely related to the communication security. The specific spectral entropy complexity calculation process is as follows:

Step 1: Remove the direct current (DC). For a chaotic pseudo-random sequence {x(n),n=0,1,2,…M×N−1} of length M×N, the following equation is used to remove the DC part, so that the spectrum can reflect the signal energy information more effectively, i.e.,

(17) $$x(n)=x(n)-\overset{-}{x}$$

where $\overset{-}{x}=\frac{1}{M\times N}\sum_{n=0}^{M\times N-1}x(n)$.

Step 2: Fourier transform. Perform a discrete Fourier transform on the sequence x(n).

(18) $$X(k)=\sum_{n=0}^{M\times N-1}x(n)e^{-j\frac{2\pi }{M\times N}\mathrm{nk}}={\sum_{n=0}^{M\times N-1}x(n)W}_{M\times N}^{\mathrm{nk}}$$

where k=0, 1, 2…M×N−1.

Step 3: Calculate the relative power spectrum. For the transformed X(k) sequence, take the first half for calculation, according to Paserval’s theorem, and calculate the power spectrum value of a certain frequency point as

(19) $$p(k)=\frac{1}{M\times N}{\left|X(k)\right|}^{2}$$

where k=0, 1, 2…M×N/2−1, and the total power of the sequence can be defined as

(20) $$p_{\mathrm{tot}}=\frac{1}{M\times N}\sum_{k=0}^{M\times N/2-1}{\left|X(k)\right|}^{2}$$

Then, the relative power spectrum probability of the sequence is $P_{k}$:

(21) $$P_{k}=\frac{p(k)}{p_{tot}}=\frac{\frac{1}{M\times N}{\left|X(k)\right|}^{2}}{\frac{1}{M\times N}\sum_{k=0}^{M\times N/2-1}{\left|X(k)\right|}^{2}}=\frac{{\left|X(k)\right|}^{2}}{\sum_{k=0}^{M\times N/2-1}{\left|X(k)\right|}^{2}}$$

Easy to know $\sum_{k=0}^{M\times N/2-1}P_{k}=1$.

Step 4: Calculate the spectral entropy. Using the relative power spectral density $P_{k}$, combined with the Shannon concept, the spectral entropy se of the signal is obtained as

(22) $$se=-\sum_{k=0}^{M\times N/2-1}P_{k}\ln P_{k}$$

where $P_{k}\ln P_{k}$ is defined to be 0 if $P_{k}$ is 0. It can be proved that the magnitude of the spectral entropy converges to ln(M×N/2). In order to facilitate the comparative analysis, the spectral entropy is normalized, and the normalized spectral entropy (SE) is obtained as

(23) $$SE(N)=\frac{se}{\ln(M\times N/2)}$$

It can be seen that when the sequence power spectrum distribution is more unbalanced, the simpler the sequence spectral entropy structure is, the more obvious oscillation laws are in the signal, and the smaller the value of SE measure obtained, i.e., the smaller the complexity is, otherwise the greater the complexity is. For spatial spectral entropy complexity maps, the darker the image color, the greater the complexity of the system, the better the pseudo-randomness of the resulting sequence and the better it can be applied in the field of encryption [31].

**Figure 12:**
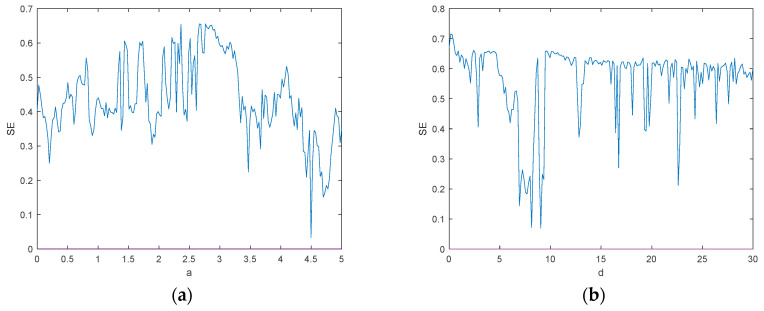
SE complexity analysis: (**a**) The spectral entropy complexity SE of $a\in \left[0,5\right]$; (**b**) the spectral entropy complexity SE of $d\in \left[0,30\right]$.

To better analyze the dynamic behavior and complexity characteristics of the chaotic system, the spectral entropy complexity SE cure of the chaotic system at $a\in \left[0,5\right]$, as shown in Figure 12a, and at $d\in \left[0,30\right]$, as shown in Figure 12b, are plotted by MATLAB 2018a. From Figure 12a,b, it can be seen that the maximum value of SE is about 0.65, which corresponds to the maximum Lyapunov exponential map, and the complexity of the system appears to have a higher value at $d\in \left[0,3.2\right]$. However, at this time, the system is in a cyclic state, and this phenomenon may be because the system has a high degree of sensitivity and small parameter variations can cause the system to be in a different state of the limit loop.

**Figure 13:**
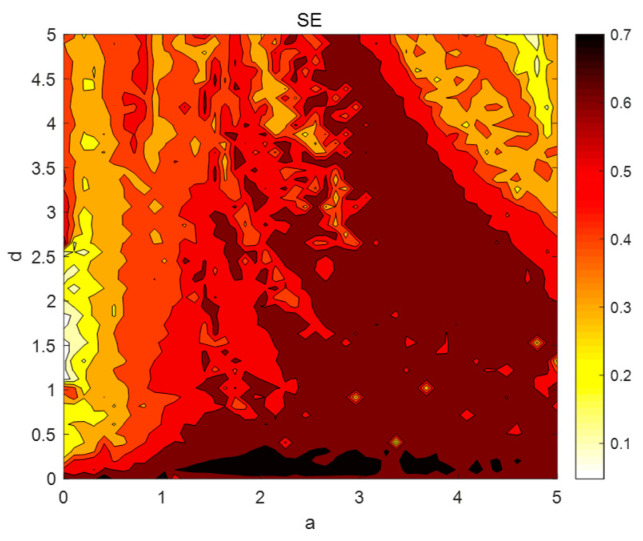
The spectral entropy complexity of $a\in \left[0,5\right]$ and $d\in \left[0,5\right]$.

The system parameters a and d are divided into 51×51 parts, where $a\in \left[0,5\right]$ and $d\in \left[0,5\right]$. The spectral entropy complexity of each point (a,d) in the parameter space is obtained by simulation, as shown in Figure 13. As can be seen from Figure 13, the complexity is mainly concentrated between $a\in \left[2.66,3.3\right]$ and $d\in \left[3.2,5\right]$. 

The complexity of the system proposed in this study is compared with the literature [32,33,34,35], and the results are shown in Table 4. Therefore, the four-dimensional chaotic system of system four has the highest complexity and has a greater advantage in practical more complex encryption environments.

## 4. Circuit Simulation

**Figure 14:**
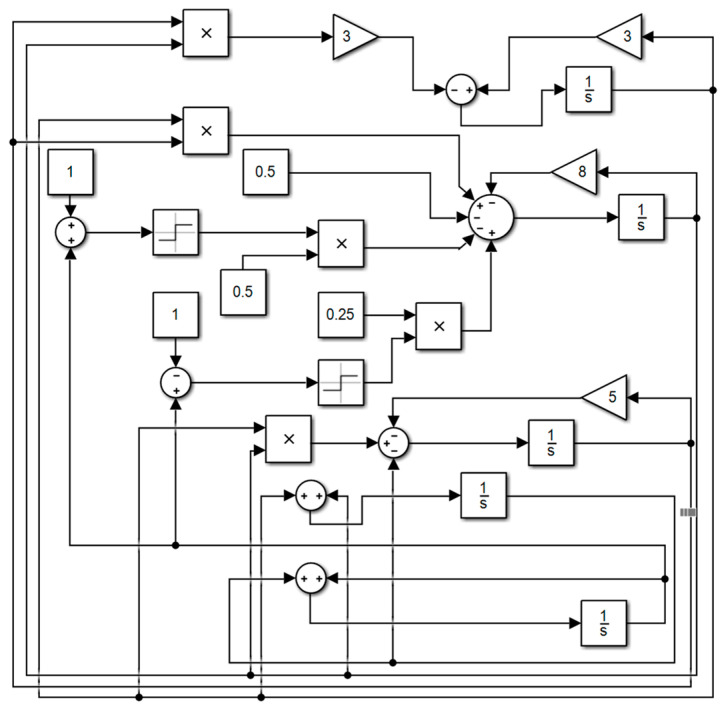
Simulink circuit diagram.

**Figure 15:**
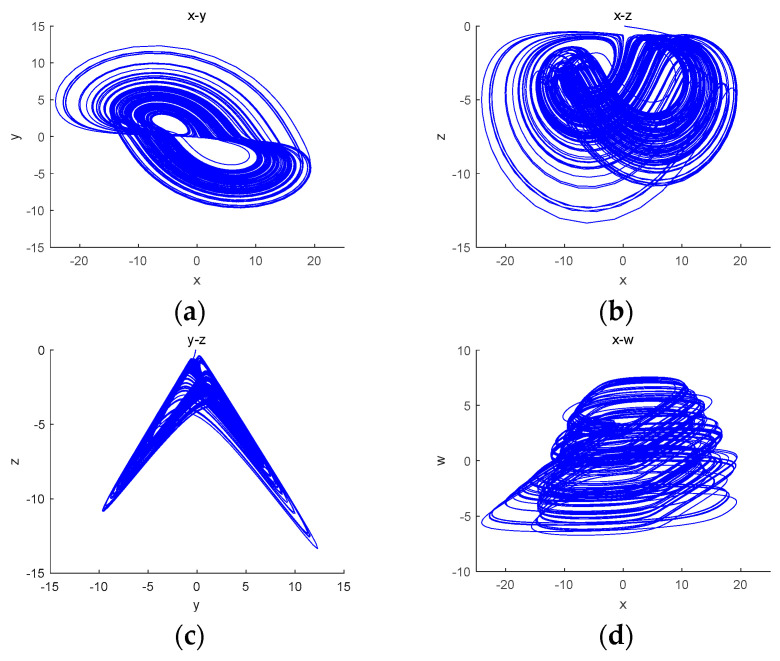
Attractor phase diagrams: (**a**) the x−y plane; (**b**) the x−z plane; (**c**) the y−z plane; (**d**) the x−w plane.

Based on the obtained model of the tri-valued memristor chaotic system, the circuit is built using the DSP Builder element in MATLAB/Simulink library [36], as shown in Figure 14, and the attractor phase diagram of the system is obtained using an oscilloscope, as shown in Figure 15. The chaotic attractor trajectories in the x−y, x−z, y−z and x−w planes obtained through numerical experiments are almost the same as the attractor phase diagrams obtained through Simulink, which verifies the correctness of this chaotic system.

## 5. Synchronized Control of Differently Structured Five-Dimensional Memristor Chaotic Systems

There are relatively few studies on the synchronization of differently structured chaotic systems based on different memristors. When high-dimensional chaotic systems containing different memristors are synchronized, it is almost impossible to predict the synchronized chaotic system at the receiver due to the different system structures at the sender and the receiver, so the synchronization of differently structured chaotic systems improves the confidentiality and security of information transmission. In this paper, we use the nonlinear feedback synchronous control method to synchronize the new fifth-order third-valued memristor chaotic system and the five-dimensional magnetically controlled memristor hyperchaotic system of the literature [37] by designing a feedback controller, which is rewritten as a five-dimensional tri-valued memristor chaotic system as the main system:

(24) $$\left\{\begin{array}{l} {\overset{•}{x}}_{1}=a_{1}x_{1}-b_{1}y_{1}z_{1}\\ {\overset{•}{y}}_{1}=-c_{1}y_{1}+x_{1}z_{1}-G(u_{1})\\ {\overset{•}{z}}_{1}=-d_{1}z_{1}+x_{1}y_{1}-w_{1}\\ \overset{•}{w_{1}}=x_{1}+y_{1}\\ {\overset{•}{u}}_{1}=w_{1}+u_{1} \end{array}\right.$$

The five-dimensional magnetically controlled memristor chaotic system of the literature [37] is rewritten as a slave system as

(25) $$\left\{\begin{array}{l} {\overset{•}{x}}_{2}=(m+nu_{2}^{2})y_{2}{-a}_{2}x_{2}+a_{2}z_{2}y_{2}+b_{2}w_{2}+v_{1}\\ {\overset{•}{y}}_{2}=-x_{2}z_{2}+c_{2}y_{2}+d_{2}x_{2}+v_{2}\\ {\overset{•}{z}}_{2}=x_{2}y_{2}-h_{2}z_{2}+y_{2}^{2}+v_{3}\\ {\overset{•}{w}}_{2}=y_{2}z_{2}-f_{2}w_{2}+v_{4}\\ {\overset{•}{u}}_{2}=g_{2}y_{2}+v_{5} \end{array}\right.$$

In Equation (25), $v_{1}$, $v_{2}$, $v_{3}$, $v_{4}$ and $v_{5}$ are synchronization controllers.

The total slave system is realized to be synchronized in different initial states, defining the error between the two systems as

(26) $$\left\{\begin{array}{l} e_{1}=x_{2}-x_{1}\\ e_{2}=y_{2}-y_{1}\\ e_{3}=z_{2}-z_{1}\\ e_{4}=w_{2}-w_{1}\\ e_{5}=u_{2}-u_{1} \end{array}\right.$$

Bringing Equations (24) and (25) into Equation (26), the error system equation is obtained as

(27) $$\left\{\begin{array}{l} {\overset{•}{e}}_{1}=me_{2}-(a_{2}-a_{1})e_{1}+b_{2}e_{4}+nu_{2}^{2}y_{2}+a_{2}y_{2}z_{2}+b_{1}y_{1}z_{1}+my_{1}-a_{2}x_{1}+b_{2}w_{1}-a_{1}x_{2}+v_{1}\\ {\overset{•}{e}}_{2}=(c_{2}-c_{1})e_{2}+d_{2}e_{1}-x_{2}z_{2}-x_{1}z_{1}+G(u_{1})+c_{2}y_{1}+d_{2}x_{1}+c_{1}y_{2}+v_{2}\\ {\overset{•}{e}}_{3}=-(h_{2}+d_{1})e_{3}-e_{4}+x_{2}y_{2}-x_{1}y_{1}+y_{2}^{2}-h_{2}z_{1}+d_{1}z_{2}+w_{2}+v_{3}\\ {\overset{•}{e}}_{4}=e_{1}+e_{2}-f_{2}e_{4}+y_{2}z_{2}-f_{2}w_{1}-x_{2}-y_{2}+v_{4}\\ {\overset{•}{e}}_{5}=g_{2}e_{2}+e_{4}+e_{5}+g_{2}y_{1}-w_{2}-u_{2}+v_{5} \end{array}\right.$$

Designed synchronous controllers are as follows:

(28) $$\left\{\begin{array}{l} v_{1}=-nu_{2}^{2}y_{2}-a_{2}y_{2}z_{2}-b_{1}y_{1}z_{1}-my_{1}+a_{2}x_{1}-b_{2}w_{1}+a_{1}x_{2}\\ v_{2}=(c_{2}-c_{1}+k_{1})e_{2}+x_{2}z_{2}+x_{1}z_{1}-G(u_{1})-c_{2}y_{1}-d_{2}x_{1}-c_{1}y_{2}\\ v_{3}=-x_{2}y_{2}+x_{1}y_{1}-y_{2}^{2}+h_{2}z_{1}-d_{1}z_{2}-w_{2}\\ v_{4}=f_{2}e_{4}-y_{2}z_{2}+f_{2}w_{1}+x_{2}+y_{2}\\ v_{5}=(k_{2}+1)e_{5}-g_{2}y_{1}+w_{2}+u_{2} \end{array}\right.$$

The controller (28) is brought into the error system (27) to obtain a linear error system written as

(29) $$\left\{\begin{array}{l} {\overset{•}{e}}_{1}=me_{2}-(a_{2}-a_{1})e_{1}+b_{2}e_{4}\\ {\overset{•}{e}}_{2}=(c_{2}-c_{1}+k_{1})e_{2}+d_{2}e_{1}\\ {\overset{•}{e}}_{3}=-(h_{2}+d_{1})e_{3}-e_{4}\\ {\overset{•}{e}}_{4}=e_{1}+e_{2}-f_{2}e_{4}\\ {\overset{•}{e}}_{5}=g_{2}e_{2}+e_{4}+(k_{2}+1)e_{5} \end{array}\right.$$

Construct the Lyapunov function as

(30) $$V=\frac{1}{2}(e_{1}^{2}+e_{2}^{2}+e_{3}^{2}+e_{4}^{2}+e_{5}^{2})$$

Derivation of Equation (28) and combining Equation (30) gives

(31) $$\begin{array}{l} \overset{•}{V}={\overset{•}{e}}_{1}e_{1}+{\overset{•}{e}}_{2}e_{2}+{\overset{•}{e}}_{3}e_{3}+{\overset{•}{e}}_{4}e_{4}+{\overset{•}{e}}_{5}e_{5}\\ =-(a_{2}-a_{1})e_{1}^{2}+(c_{2}-c_{1}+k_{1})e_{2}^{2}-(h_{2}+d_{1})e_{3}^{2}-f_{2}e_{4}^{2}+(k_{2}+1)e_{5}^{2} \end{array}$$

When $k_{1}<c_{1}-c_{2}$, $k_{2}<-1$, $\overset{•}{V}<0$, the error system (27) converges exponentially to the global equilibrium point and the error system tends to zero, i.e., the two differently structured systems are synchronized.

**Figure 16:**
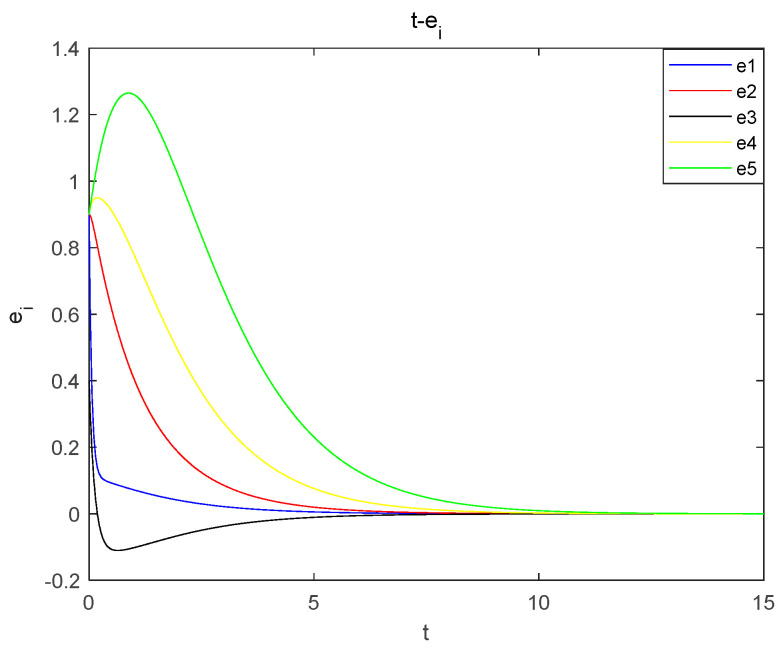
Nonlinear feedback synchronization error plot.

Experimental simulation of the proposed nonlinear feedback synchronous control is carried out, the initial value of the master system is (0.1, 0.1, 0.1, 0.1, 0.1) and the initial value of the slave system is (1, 1, 0.5, 1, 1). Taking the $k_{1}=-3$, $k_{2}=-2$ of the controller, the error curves are obtained as shown in Figure 16, from which it can be seen that the master and slave systems are synchronized at t=13. This verifies the correctness and validity of the design of the controller and proves that the newly proposed chaotic system can be applied in the field of encryption.

## 6. Variable Parameter Color Image Encryption Scheme

### 6.1. Encryption and Decryption Program

Traditional chaos-based encryption regimes generally use fixed parameters, and the analysis and deciphering of system tracks make the algorithms less secure. Therefore, a dynamic parameter encryption scheme is proposed based on the framework system of diffusion, disruption and DNA encryption algorithms by taking advantage of the parameter tunability of five-dimensional tri-valued memristor chaotic systems. The process is described below:

Step 1: Determining the key. The three color components of the plaintext color image P are transformed into one-dimensional vectors R, G and B. The pixel averages of their components are found separately and combined as the key and as the initial value of the chaotic system, as shown in Equations (32) and (33) below.

(32) $$\left\{\begin{array}{l} avR=\frac{\sum_{x=1}^{M}\sum_{y=1}^{N}R(x,y)}{M\times N}\\ avG=\frac{\sum_{x=1}^{M}\sum_{y=1}^{N}G(x,y)}{M\times N}\\ avB=\frac{\sum_{x=1}^{M}\sum_{y=1}^{N}B(x,y)}{M\times N} \end{array}\right.$$

(33) $$\left\{\begin{array}{l} x0=avR\\ y0=avG\\ z0=avB\\ w0=(avR+avG)/2\\ u0=(avG+avB)/2 \end{array}\right.$$

Step 2: Chunking. M×N chunking operation is performed on the color image P of size M×N, which is uniformly divided into images of size M/4×N/4. The chunked images are transformed into one-dimensional vectors, denoted as $P_{1}$, $P_{2}$, $P_{3}$ and $P_{4}$, respectively.

Step 3: Diagonal block encryption methods $P_{1}$ and $P_{4}$.

1. Generate a chaotic pseudo-random sequence $S_{i}$ of length M×N using chaotic system, discard the first 1500 points of the transition state and perform additive modulo left-shift diffusion operation on a component of the image $P_{i}$ obtained from the chunking. Convert the component into a gray-scale image with eight bits for each pixel point. The lower three bits of any data range from zero to seven as the effective range of cyclic shifting of a pixel point’s data, then the plaintext after the cyclic left shift of the data and the chaotic sequence are added and each byte is summed and modeled to obtain the encrypted sequence $C_{i}$. The computation is shown in Equation (34).
  (34) $$C_{i}=(C_{i-1}+S_{i}+P_{i})\mathrm{mod}256<<<{LSB}_{3}(C_{i-1})$$
  where $C_{i}$ is a ciphertext sequence, $S_{i}$ is a chaotic sequence, $P_{i}$ is a plaintext vector and $<<<{LSB}_{3}$ is a cyclic left shift to the lowest three bits of the data.
2. From the dynamical behavior of the new system, it can be seen that the complexity of the system is higher when parameter d=9.648. Take this time to generate a pseudo-random sequence M/4×N/4 of length $S_{12}$. Perform a modulo left-shift diffusion operation on the $P_{1}$ component of image G obtained from the chunking to obtain the encrypted sequence $C_{1G}$.
3. When parameter a=2.764, the complexity of the system is the highest. Take this time to produce a pseudo-random sequence M/4×N/4 of length $S_{13}$. The repeated pseudo-random numbers in $S_{13}$ are eliminated and only the first occurrence of the value is retained, and then the elements in the set {1,2,…,MN/16} that do not appear in $S_{13}$ are ranked from smallest to largest. Then, $P_{1}(S_{i})$ is swapped with $P_{1}(S_{\frac{MN}{16}-i+1})$ to change the position and component B of image $P_{1}$ obtained from the chunking is dislocated without repetition to obtain the encrypted sequence $C_{1B}$.

Similarly, the R, G and B of the chunked $P_{4}$ are encrypted in the same way as $P_{1}$.

Step 4: Diagonal block encryption methods $P_{2}$ and $P_{3}$.

1. Keeping the system parameters unchanged and making the system parameters d=9.648, the components R and G of $P_{2}$ are encrypted without repetitive disruption, respectively, in the same way as in Step 3 of 3.
2. Let parameter a=2.764, generate a pseudo-random sequence $S_{23}$ and perform a modulo left-shift diffusion operation on the component B of $P_{2}$, encrypted in the same way as in Step 3 of 1.

Similarly, the R, G and B of the chunked $P_{3}$ are encrypted in the same way as $P_{2}$.

Step 5: Combining the images. The sub-blocks that have been diffused and encrypted are recombined to obtain image E.

Step 6: DNA encryption.

1. Separate the components R, G and B of image E.
2. Keeping the parameters unchanged, sequences {x}, {y}, {z}, {w} and {u} of length M×N are generated and sequence {u} is transformed into a two-dimensional matrix Q, which is used for DNA operations with component R of image E.
  (35) Q=reshape(U,M,N)
3. Sequence {x} determines the encoding mode of the DNA of each component R of image E, and sequence {y} determines the DNA encoding mode of matrix Q. There are a total of eight modes of DNA encoding and decoding, which need to be transformed into {x} and {y}. After the transformation, the value of each sequence is a random integer from one to eight, and the transformation equation is shown in Equation (36):
  (36) $$\left\{\begin{array}{l} X=\mathrm{mod}(floor(X\times 10^{4}),8)+1\\ Y=\mathrm{mod}(floor(Y\times 10^{4}),8)+1 \end{array}\right.$$
4. The decoding of DNA is determined by sequence {w}. DNA decoding is the inverse process of DNA coding, and there are eight ways of decoding, i.e., decoding A, G, C and T into specific binary values.
  (37) $$W=\mathrm{mod}(floor(W\times 10^{4}),8)+1$$
5. There are three algorithms for defining DNA, and the DNA algorithm is determined by the system-generated sequence {z}. If $Z_{i}=0$, additive, if $Z_{i}=1$, subtractive and if $Z_{i}=2$, different-or. Sequence {z} is transformed according to Equation (38) into integers ranging from zero to three.
  (38) $$Z=\mathrm{mod}(floor(Z\times 10^{4}),3)$$

Similarly, make d=9.648 and repeat Step 6 for DNA encryption of component G; make a=2.764 and repeat Step 6 for DNA encryption of component B.

Step 7: Combining the three 2D matrices encrypted by DNA into a 3D matrix to obtain the encrypted image.

**Figure 17:**
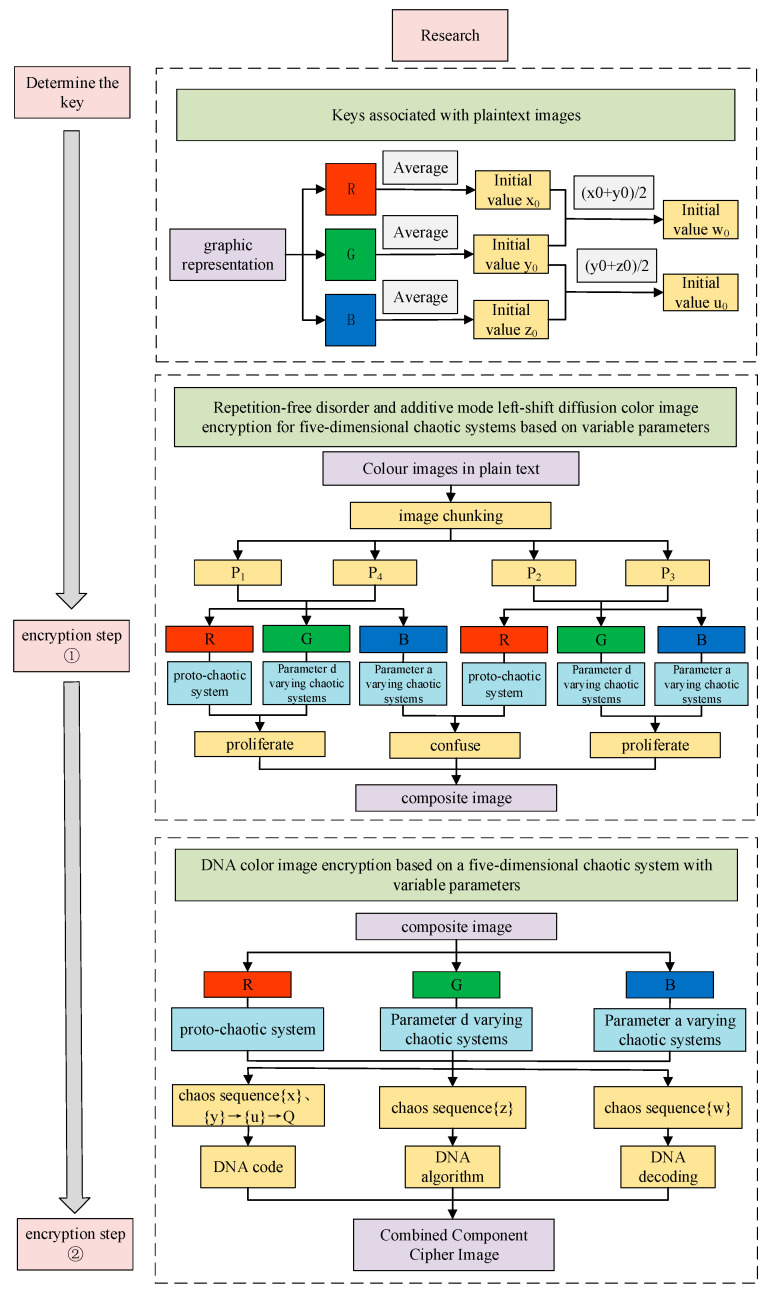
Encryption flowchart.

The encryption flowchart is shown in Figure 17.

The encryption algorithms used are all reversible, so the decryption process is the inverse of the encryption process. The encrypted image is subjected to DNA encoding, DNA inverse operation, DNA decoding, inverse scrambling and inverse diffusion in order to obtain the decrypted image. The decrypted image can be obtained. The specific decryption steps are as follows:

Step 1: Determining the key. Same as the first step of the encryption process.

Step 2: DNA decoding. The reverse process of DNA coding is known as DNA decoding.

1. Separate the components R, G and B of ciphertext image E.
2. Keeping the parameters unchanged, sequences {x}, {y}, {z}, {w} and {u} of length M×N are generated, and sequence {u} is transformed into a two-dimensional matrix Q, which is used for DNA operations with component R of image E.
  (39) Q=reshape(U,M,N)
3. Sequence {x} determines the decoding method of DNA for each component R of image E, and sequence {y} determines the decoding method of DNA for matrix Q. There are eight ways to encode and decode DNA, which need to transform {x} and {y}. The value of each sequence after transformation is a random integer from one to eight, and transformation Equation (40) is as follows:
  (40) $$\left\{\begin{array}{l} X=\mathrm{mod}(floor(X\times 10^{4}),8)+1\\ Y=\mathrm{mod}(floor(Y\times 10^{4}),8)+1 \end{array}\right.$$
4. Sequence {w} determines how the DNA is encoded, and there are eight ways to decode it, i.e., A, G, C and T are encoded into specific binary values.
  (41) $$W=\mathrm{mod}(floor(W\times 10^{4}),8)+1$$
5. There are three algorithms for defining DNA, and the DNA algorithm is determined by the system-generated sequence {z}. If $Z_{i}=0$, subtraction is used; if $Z_{i}=1$, addition is used; and if $Z_{i}=2$, inverse heteroscedasticity is used. Sequence {z} is transformed into an integer in the range zero to three by the transformation of Equation (42).
  (42) $$Z=\mathrm{mod}(floor(Z\times 10^{4}),3)$$

Similarly, let d=9.648, repeat Step 6 for DNA decryption of component G; let a=2.764, repeat Step 6 for DNA decryption of component B.

Step 3: Combining the 2D vectors of the three-component RGB after performing DNA decryption to obtain color cipher image C.

Step 4: Chunking. The chunking operation is performed on the color ciphertext image C of size M×N, which is uniformly divided into images of size M/4×N/4. The chunked image is converted into one-dimensional vectors, denoted as $C_{1}$, $C_{2}$, $C_{3}$ and $C_{4}$, respectively.

Step 5: Diagonal block decryption method $C_{1}$ and $C_{4}$.

1. Using the chaotic system to generate a chaotic pseudo-random sequence $S_{i}$ of length M/4×N/4, discard the first 1500 points of the transition state, carry out the reverse additive modulo left-shift diffusion operation on a certain component of the ciphertext image C obtained from the chunking and transform the component into a gray-scale image with eight bits for each pixel point. The low three bits of any piece of data are taken as the value range of 0~7 as the effective range of cyclic shifting of a pixel point’s data, and then after the cyclic right shift the plaintext data and the chaotic sequence for the addition operation, each byte is added and modulo to obtain the decrypted sequence $P_{i}$. Equation (43) is as follows:
  (43) $$P_{i}=(2\times 256+C_{i}-C_{i-1}-S_{i})\mathrm{mod}256>>>LSB_{3}(C_{i-1})$$
  where $C_{i}$ is a ciphertext sequence, $S_{i}$ is a chaotic sequence, $P_{i}$ is a plaintext vector and $>>>LSB_{3}$ is a cyclic right shift of the lowest three bits of the data.
2. From the dynamical behavior of the new system, it can be seen that the complexity of the system is higher when parameter d=9.648. Taking this time, a pseudo-random sequence $S_{12}$ of length M/4×N/4 is generated and an inverse modulo left-shift diffusion operation is performed on the component $P_{1}$ of image G obtained from the chunking to obtain decrypted sequence $C_{1G}$.
3. When parameter a=2.764, the complexity of the system is the highest. Take this time to produce a pseudo-random sequence $S_{13}$ of length M/4×N/4. The repeated pseudo-random numbers in $S_{13}$ are eliminated and only the first occurrence of the value is retained, and then the elements in the set $\left\{1,\;2,\;\ldots ,\;\frac{MN}{16}\right\}$ that do not appear in $S_{13}$ are ranked from smallest to largest. Then, $C_{1}(S_{\frac{MN}{16}-i+1})$ and $C_{1}(S_{i})$ are swapped for the position of components B of image $C_{1}$ obtained from the chunking to perform a repetition-free disarray and to obtain the decrypted sequence $C_{1B}$.

Similarly, the R, G and B of the chunked $C_{4}$ are decrypted in the same way as image $C_{1}$.

Step 6: Decrypting the diagonal blocks way $C_{2}$ and $C_{3}$.

1. Keeping the system parameters unchanged and letting system parameters d=9.648, the inverse non-repetitive disambiguation operation is performed on component G and component R of $C_{2}$, respectively, decrypted in the same way as in the third step (3).

Let parameter a=2.764, generate a pseudo-random sequence $S_{23}$ and perform a reverse modulo left-shift diffusion operation on component B of $C_{2}$. The decryption in the same way as in Step 5 of 3.

Step 7: Combining the images. The sub-blocks that have been diffused and decrypted are recombined to obtain original image P.

**Figure 18:**
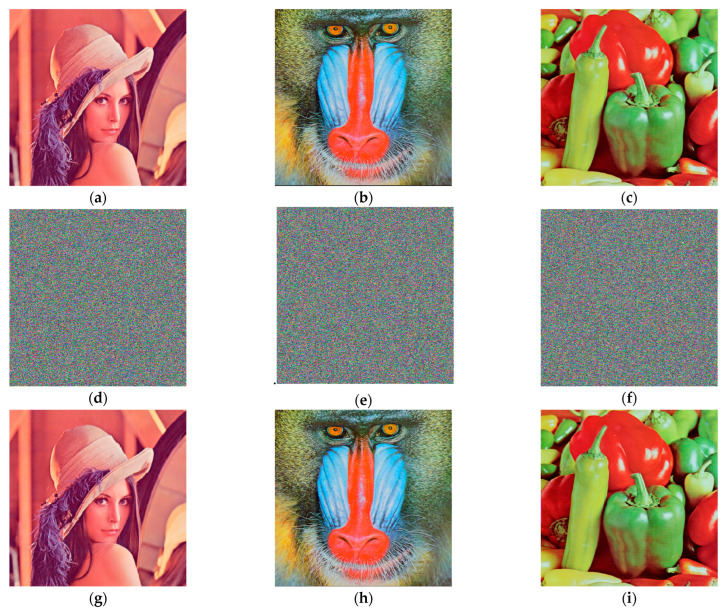
Original images, encrypted images and decrypted images: (**a**) the Lena original image; (**b**) the Baboon original image; (**c**) the Peppers original image; (**d**) the Lena encrypted image; (**e**) the Baboon encrypted image; (**f**) the Peppers encrypted image; (**g**) the Lena decrypted image; (**h**) the Baboon decrypted image; (**i**) the Peppers decrypted image.

Using the above proposed variable parameter encryption and decryption scheme, the encryption and decryption operations are carried out on RGB color Lena, Baboon and Peppers images of size 512×512 pixels using MATLAB 2018a, and the encryption results are shown in Figure 18. It is difficult to see the information of the original image from the encrypted images, and the decrypted images do not differ much from the original images, which proves that the encryption and decryption algorithm is feasible.

### 6.2. Performance Analysis

#### 6.2.1. Histogram Tests

**Figure 19:**
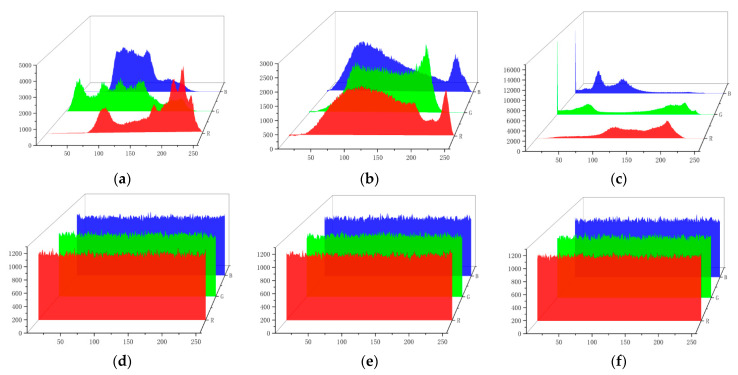
Histogram tests: (**a**) the Lena plaintext image; (**b**) the Baboon plaintext image; (**c**) the Peppers plaintext image; (**d**) the Lena encrypted image; (**e**) the Baboon encrypted image; (**f**) the Peppers encrypted image.

The distribution of the number of pixel points can be seen from the histograms. The histograms of the RGB components of the color Lena image, Baboon image and Peppers image are shown in Figure 19. The histogram of the plaintext image falls and falls, while the encrypted images have a uniform distribution, based on which the encryption algorithm effectively hides the statistical features of the original image, and it is difficult for the attacker to predict the original image using statistical analysis.

#### 6.2.2. Pixel Distributions

**Figure 20:**
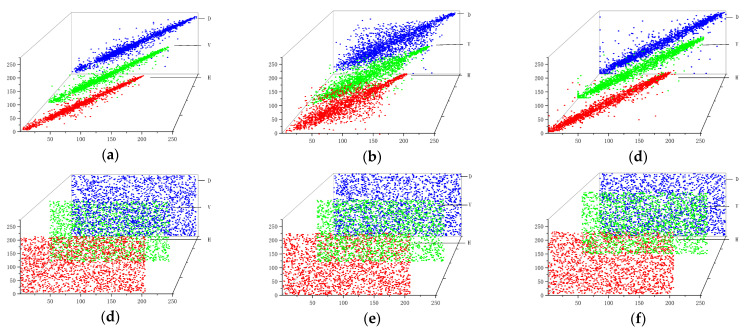
Pixel distributions: (**a**) the Lena plaintext image; (**b**) the Baboon plaintext image; (**c**) the Peppers plaintext image; (**d**) the Lena encrypted image; (**e**) the Baboon encrypted image; (**f**) the Peppers encrypted image.

The correlation coefficient is a quantitative expression of the image pixel correlation, and to prevent statistical attacks, the correlation needs to be reduced to hide the statistical information of the plaintext. The distributions of neighboring pixels in the horizontal, vertical and diagonal directions for the Lena, Baboon and Peppers plaintext images and the encrypted images are shown in Figure 20. Taking Lena image as an example, the correlation coefficients of this paper’s algorithm are compared with those of the algorithms in the literature [38,39,40], and the results are shown in Table 5. The correlation of neighboring pixel points of plaintext images is stronger and the pixel points are concentrated in the nearby distribution, while the correlation of encrypted images encrypted by the encryption algorithm is close to zero and the distribution of pixel points is more randomized and close to a uniform distribution, which is more conducive to hide the plaintext information.

#### 6.2.3. Information Entropy

The information entropy reflects the uncertainty of the image information, the greater the entropy (close to eight), the greater the uncertainty and the less the visible information, calculated as

(44) $$H=-\sum_{i=0}^{L-1}p(x_{i})\log_{2}p(x_{i})$$

where $x_{i}$, i=0, 1, 2…L−1 denote the gray value of the image, and ${p(x}_{i})$ denote the probability of occurrence of the gray values.

The distribution of gray values of the three channels RGB of the color image is counted using the above equation, and the percentage of gray values of the component RGB, i.e., the probability of each gray value, is calculated. The final information entropy of the color image is calculated by calculating the average of the three channels as the information entropy of the color image and will be compared with the literature [41,42,43], as shown in Table 6. In this paper, the information entropy is closer to eight, so the algorithm can be more resistant to entropy attacks.

#### 6.2.4. Differential Attack

Differential attack is a powerful crypt analysis method that focuses on inferring the key by using the difference information between plaintext and ciphertext pairs. The number of pixels change rate (NPCR) and Unified Average Changing Intensity (UACI) are usually used to evaluate whether an algorithm can resist differential attacks. In general, the closer the NPCR is to 99.6094% and the UACI is to 33.4635% [44], the better the performance of the encryption algorithm.

A pixel is randomly selected from the original image, and one of the pixel values is modified according to Equation (45) to acquire image P. The original image and modified image P are encrypted using the algorithm proposed in this paper to obtain cipher images $P_{1}$ and $P_{2}$. The difference between $P_{1}$ and $P_{2}$ is quantified by NPCR and UACI, and the NPCR and UACI values are computed as shown in Equations (46)–(48).

(45) value=mod(value+1,256)

(46) $$NPCR(P_{1},P_{2})=\frac{1}{MN}\sum_{i=1}^{M}\sum_{j=1}^{N}D(i,j)\times 100\%$$

(47) $$UACI(P_{1},P_{2})=\frac{1}{MN}\sum_{i=1}^{M}\sum_{j=1}^{N}\frac{\left|P_{1}(i,j)-P_{2}(i,j)\right|}{255}\times 100\%$$

(48) $$D(i,j)=\left\{\begin{array}{l} 0,P_{1}(i,j)=P_{2}(i,j)\\ 1,P_{1}(i,j)\neq P_{2}(i,j) \end{array}\right.$$

where $P_{1}$ is the original cipher image, $P_{2}$ is the cipher image with one pixel point changed, $P_{1}(i,j)$ is any position of the $P_{1}$ image, $P_{2}(i,j)$ is any position of the $P_{2}$ image, M and N are the image size, when $P_{1}(i,j)$ is equal to $P_{2}(i,j)$, the value of D(i,j) is 0 and when $P_{1}(i,j)$ is not equal to $P_{2}(i,j)$, the value of D(i,j) is 1.

The values of NPCR and UACI of Lena image are calculated using the above equation and compared with the literature [45,46] methods. The specific results are shown in Table 7, and the encryption algorithm in this paper is closer to the ideal values compared to the other literature. The difference in NPCR is 0.0009, while the difference in the literature [45] is 0.001, the difference in the literature [46] is 0.0013 and the difference between this paper and the ideal value of NPCR is the smallest; the difference in UACI is 0.0057, while the difference in the literature [45] is 0.0122, the difference in the literature [46] is 0.0157 and the difference between this paper and the ideal value of UACI is the smallest. It is proved that the encryption scheme has good sensitivity and high resistance to differential attacks. The algorithm proposed in this paper is parametrically dynamic compared to the literature [45,46], and the proposed DNA encryption scheme is parametrically dynamic, which greatly improves the ability to be deciphered.

In this paper, we propose a dynamic variable parameter encryption scheme based on more complex new five-dimensional tri-valued memristor chaotic systems, combining repeat-free disruption and additive mode left-shift diffusion methods, as well as DNA encryption methods. A differential attack is one of the more difficult attacks to resist among all the attacks, and the algorithm proposed in this paper can resist differential attacks better as compared to other encryption methods. This result is mainly because the parameters chosen for the encryption scheme in this paper are dynamically changing, and the attacker cannot simply perform a differential attack by analyzing a fixed parameter, which greatly increases the difficulty of deciphering and thus improves the security of the crypto system. This is the biggest advantage and difference in the dynamic variable parameter encryption scheme proposed in this paper over other advanced encryption methods.

#### 6.2.5. Noise Attack

Strong encryption algorithms are tolerant to noise attacks to protect the image. To test the noise immunity of the encryption scheme, the encryption performance is tested in this paper by adding salt-and-pepper noise (SPN) and Gaussian noise (GN). The peak signal-to-noise ratio (PSNR) is used to measure the ability to resist noise attacks; in general, the larger the PSNR, the stronger the noise resistance of the encryption algorithm.

**Figure 21:**
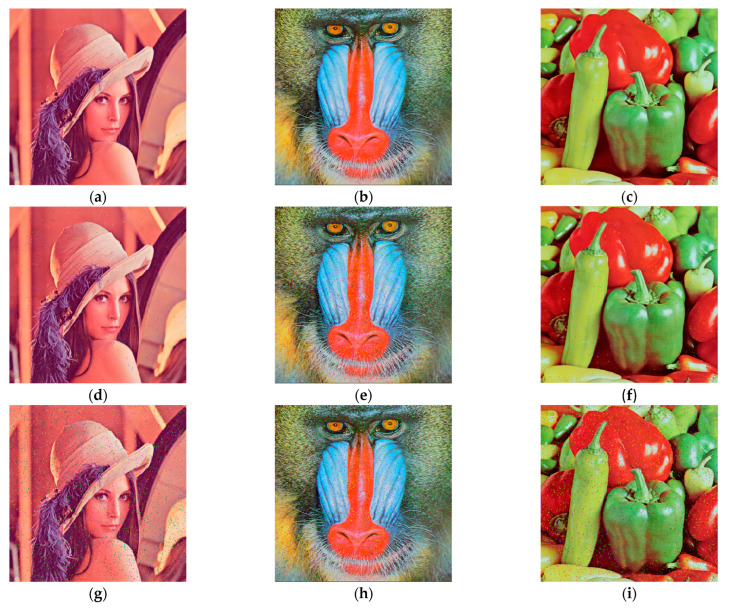
Decrypted images with different levels of salt-and-pepper noise added: (**a**) the Lena image with 0.01% salt-and-pepper noise; (**b**) the Baboon image with 0.01% salt-and-pepper noise; (**c**) the Peppers image with 0.01% salt-and-pepper noise; (**d**) the Lena image with 0.1% salt-and-pepper noise; (**e**) the Baboon image with 0.1% salt-and-pepper noise; (**f**) the Peppers image with 0.1% salt-and-pepper noise; (**g**) the Lena image with 1% salt-and-pepper noise; (**h**) the Baboon image with 1% salt-and-pepper noise; (**i**) the Peppers image with 1% salt-and-pepper noise.

Salt-and-pepper noise is a common type of noise caused by variations in the intensity of signal pulses. When salt-and-pepper noise with densities of 0.01%, 0.1% and 1% is added to the Lena image, Baboon image and Peppers image for testing, the decrypted images obtained are shown in Figure 21. The PSNR values of the decrypted images containing densities of 0.01%, 0.1% and 1% salt-and-pepper noise obtained by numerical computation using MATLAB 2018a are shown in Table 8. When affected by salt-and-pepper noise with a density of 0.01%, the PSNR value of the decrypted images is greater than 31 dB, indicating that the quality of the decrypted images is better, even if there are distortions that cannot be detected by the naked eye. The PSNR value of the decrypted images is greater than 25 dB when affected by salt-and-pepper noise with a density of 0.1%, indicating that the decrypted images are of average quality with distortion. When subjected to salt-and-pepper noise with a density of 1%, the PSNR value of the decrypted images is greater than 16 dB and the presence of variability from the original images are already evident. Thus, it is proved that the algorithm can resist a certain degree of noise attack with high robustness.

**Figure 22:**
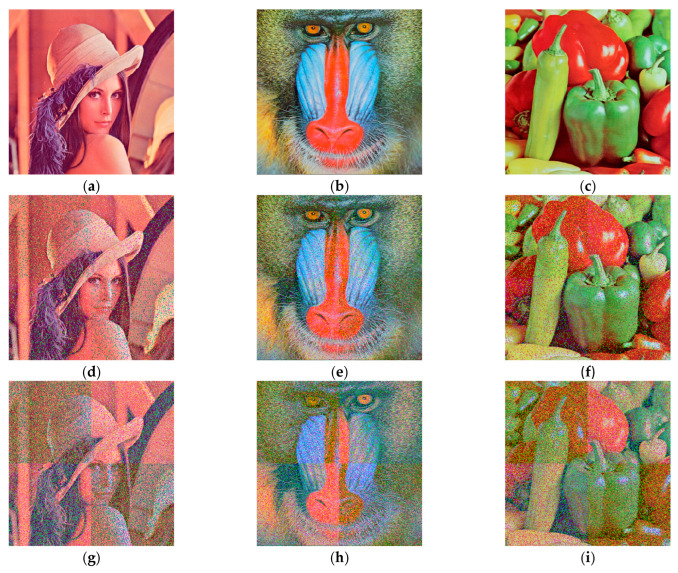
Decrypted images with different levels of Gaussian noise added: (**a**) the Lena decrypted image of Gaussian noise with variance $1\times 10^{-10}$; (**b**) the Baboon decrypted image of Gaussian noise with variance $1\times 10^{-10}$; (**c**) the Peppers decrypted image of Gaussian noise with variance $1\times 10^{-10}$; (**d**) the Lena decrypted image of Gaussian noise with variance $1\times 10^{-6}$; (**e**) the Baboon decrypted image of Gaussian noise with variance $1\times 10^{-6}$; (**f**) the Peppers decrypted image of Gaussian noise with variance $1\times 10^{-6}$; (**g**) the Lena decrypted image of Gaussian noise with variance $2\times 10^{-6}$; (**h**) the Baboon decrypted image of Gaussian noise with variance $2\times 10^{-6}$; (**i**) the Peppers decrypted image of Gaussian noise with variance $2\times 10^{-6}$.

Gaussian noise is a class of noise whose probability of occurrence obeys a Gaussian distribution and noise with a random depth of noise occurs at each pixel point of an image affected by Gaussian noise. Keeping the mean value as 0, when Gaussian noises with variance $1\times 10^{-10}$, variance $1\times 10^{-6}$ and variance $2\times 10^{-6}$ are added to Lena image, Baboon image and Peppers image for testing, the decrypted images obtained are shown in Figure 22. The PSNR values of the decrypted images obtained for Gaussian noise with different variances and means are shown in Table 9. When the effect of Gaussian noise with variance $1\times 10^{-10}$ is added, the PSNR values of the decrypted images are greater than 33 dB, the quality of the decrypted images are better and the distortion is not detectable by the naked eye. When the effect of Gaussian noise with variance $1\times 10^{-6}$ is added, the PSNR values of the decrypted images are greater than 10 dB, indicating that the decrypted images are already distorted. When the effect of Gaussian noise with variance $2\times 10^{-6}$ is added, the PSNR values of the decrypted images are greater than 6.9 dB, which can largely reveal the outline of the images and the general information of the subject, but the encryption algorithm is not as good at resisting Gaussian noise as it is at resisting salt-and-pepper noise.

In summary, the increase in the intensity of salt-and-pepper noise and Gaussian noise gradually affects the quality of the decrypted image, but the main features of the image are still recognizable. This indicates that the proposed dynamic encryption scheme can resist the attack of salt-and-pepper noise and Gaussian noise to a certain extent with high robustness.

#### 6.2.6. Geometric Attack

**Figure 23:**
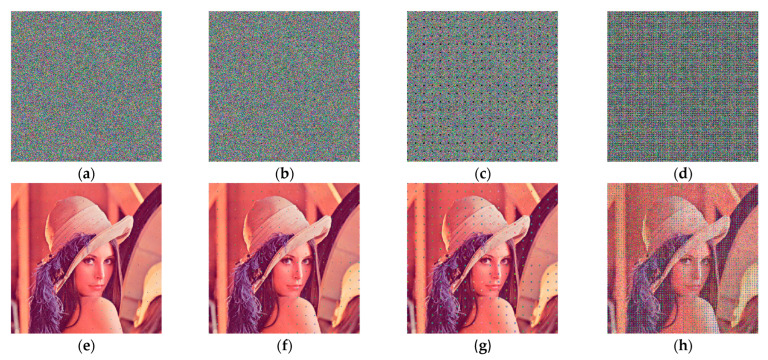
Lena encrypted and decrypted images under cropping attack: (**a**) Cropping 0.0976% of Lena encrypted image; (**b**) cropping 0.391% of Lena encrypted image; (**c**) cropping 1.56% of Lena encrypted image; (**d**) cropping 6.25% of Lena encrypted image; (**e**) cropping 0.0976% of Lena decrypted image; (**f**) cropping 0.391% of Lena decrypted image; (**g**) cropping 1.56% of Lena decrypted image; (**h**) cropping 6.25% of Lena decrypted image.

The Lena image is used as an experimental object to verify the resistance of this encryption algorithm to geometric attacks. The most common attack means of geometric attacks is the cropping attack, so the cropping attack is used as an example to decrypt the cipher image that has been missing information blocks with the same and correct key as the encryption process. The encrypted images are cropped uniformly, and the cropping sizes are 0.0976%, 0.391%, 1.56% and 6.25% of the original images, respectively. The resulting cropped encrypted images as well as decrypted images are shown in Figure 23. The main information of the decrypted images can still be recognized, and the recovery of the decrypted images becomes weaker as the clipped area increases. Therefore, the algorithm is resistant to cropping.

#### 6.2.7. Image Quality Analysis

To test and measure the quality of encrypted and decrypted images, PSNR and Structural Similarity (SSIM) are quantitatively analyzed, in which the higher the value of PSNR, the better the quality of the reconstructed image and the higher the degree of restoration of the image information. The closer the value of SSIM is to one means the more similar the two images are. Lena image, Baboon image and Pepper image are selected and analyzed to calculate the PSNR value and SSIM value of the encrypted and decrypted images, as shown in Table 10. The PSNR values of all three groups of encrypted images are less than 4.1 dB and the SSIM values are less than 0.0094, which indicate that the encrypted images are so different from the original images that the useful information of the original image can not be directly observed and the encryption effect is better. The PSNR values of the three groups of decrypted images are all greater than 33 dB and the SSIM values are greater than 0.9993, indicating that the decrypted images are very similar to the original images, visually close to the plaintext images and the quality of the decrypted images are more satisfactory.

#### 6.2.8. Key Space

**Figure 24:**
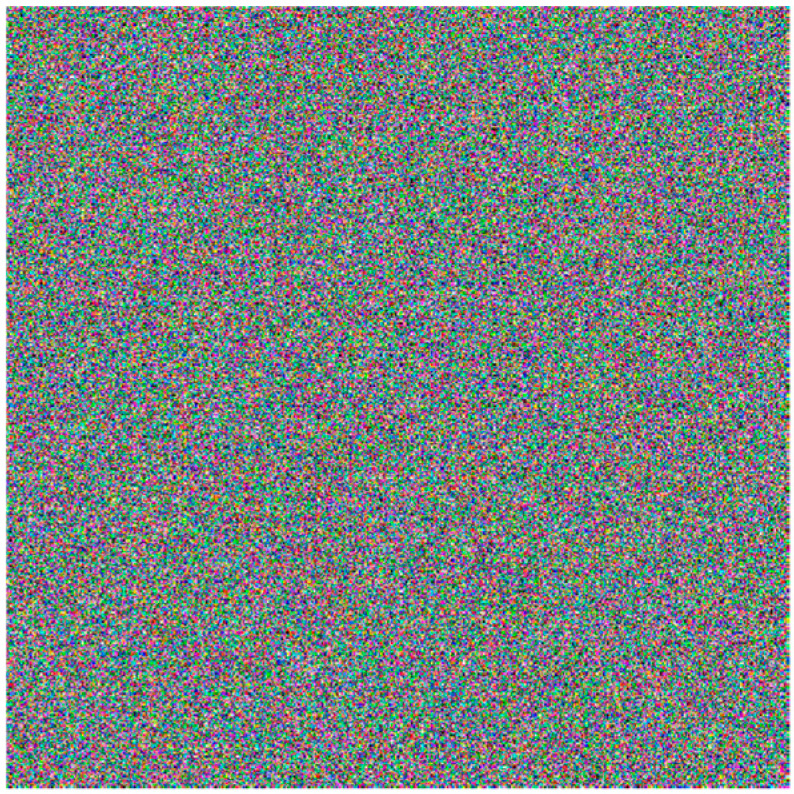
Error key decryption diagram.

This algorithm takes the pixel average of the plaintext image as the initial value of the new chaotic system and also as the key for encryption, and the accuracy of the initial value can reach $10^{-15}$. Therefore, the size of the key space is $10^{75}$. The value is much larger than $2^{128}$. It can be regarded that the key space is a secure key space, which is able to resist the exhaustive attack. When any one of the fifteen-bit error keys are changed for decryption, the decrypted image obtained is shown in Figure 24, which shows that the key based on the dynamic parameter encryption scheme has a certain degree of sensitivity.

### 6.3. Analysis of Application Scenarios

For the current more complex encryption environment, the encryption performance, key management and encryption quality and other requirements are higher, such as military, medical, financial and other fields, which often need to use very strong encryption algorithms. The dynamic encryption algorithm can resist statistical attacks, differential attacks, noise attacks and shear attacks, the ciphertext information of the neighboring pixel points are more uniformly distributed, from which the original plaintext information can not be observed. The image quality is higher and the key space is larger, which can well ensure the confidentiality, integrity and availability of information. Therefore, it can be applied to military, medical, financial and other fields.

Encryption of medical data in healthcare organizations to protect patient privacy and data confidentiality from unauthorized access and leakage, where medical images, medical records, prescriptions, etc., can be stored or transmitted, can be encrypted using this encryption technology. In military institutions where the security of sensitive data such as operational plans, intelligence information, map data, etc., needs to be protected, the encryption scheme can be used by military command, control and intelligence departments to protect the confidentiality of sensitive information against eavesdropping and theft by hostile forces. The encryption technology can help financial institutions protect the confidentiality of large amounts of sensitive customer information and financial data, including personal identification information, account balances, transaction records, etc., against data leakage and illegal access.

## 7. Summary and Outlook

In this paper, a new five-dimensional memristor chaotic system with rich dynamical behavior is firstly constructed by using a tri-valued memristor. Compared with the previous chaotic systems, the new system improves the complexity and exhibits strong sensitivity to parameter variations, with the existence of not only the periodic limit ring and chaotic attractor depending on the system coupling parameter, but also the variable wing phenomenon depending on the system parameter and the memristor parameter. In addition to this, the system has two different types of transient chaotic phenomena, chaos-periodic and chaos-quasi-periodic depending on the coupling parameter of the system and the memristor parameter. Secondly, the circuits of the new five-dimensional memristor chaotic system are constructed by using the simulation devices in the Simulink platform, which are consistent with the numerical simulation results and verify the correctness of the new system. Finally, a variable parameter color image encryption method is proposed based on this chaotic system, which associates the values of the three component pixel points of the color image with the key according to the principle of one image, one key, to enhance the sensitivity and security of the plaintext and the key. The sequence generated by the parametric dynamic characteristics of the new system is used to encrypt the three color components of the RGB of the color image after chunking by combining additive mode left-shift diffusion and no-repeat disambiguation. DNA encoding and operations are performed on the 2D matrix of the combined color image, and the three channels are finally combined to obtain the encrypted image. In addition, the security analysis of the encrypted image shows that the algorithm can effectively counter the statistical analysis attack, differential attack, noise attack and cropping attack, the information entropy of the encrypted image is close to eight, the correlation coefficient is close to zero, the PSNR values of the decrypted image quality are all greater than 33 dB and the SSIM values are all greater than 0.9990, which verify the security and effectiveness of the algorithm. However, the algorithm is weak against filtering attacks and image compression attacks, and the algorithm will be optimized for these two parts subsequently. In the future, hardware implementations based on this encryption scheme could be considered for implementation in areas such as medical imaging, military encryption and financial data protection.

## Figures and Tables

**Table 1:** State of the system corresponding to the 0–1 test value $K_{c}$.

$K_{c}$	System Status
0	periodicity
1	chaotic

**Table 2:** Attractor categories and numbers corresponding to different values of parameter a.

System Parameter a	Attractor Subcategory	Corresponding Number	System Parameter a	Attractor Subcategory	Corresponding Number
0.3	Cycle 3 limit ring	Figure 5a	0.7	Cycle 6 limit ring	Figure 5b
0.8	Cycle 8 limit ring	Figure 5c	0.9	Cycle 9 limit ring	Figure 5d
1.2	Cycle 2 limit ring	Figure 5e	1.6	Cycle 4 limit ring	Figure 5f
2.8	chaotic	Figure 5g	4.2	quasi-cycle	Figure 5h
4.8	quasi-cycle	Figure 5i			

**Table 3:** System states corresponding to different values of parameter $b_{0}$.

$\mathrm{Parameter}\;b_{0}$ Interval	System Status	$\mathrm{Parameter}\;b_{0}$ Interval	System Status
$b_{0}\in \left[0,1.22\right]$	chaotic	$b_{0}\in \left[1.22,1.56\right]$	periodicity
$b_{0}\in \left[1.56,2.14\right]$	chaotic	$b_{0}\in \left[2.14,2.39\right]$	periodicity
$b_{0}\in \left[2.39,2.81\right]$	chaotic	$b_{0}\in \left[2.81,3.94\right]$	quasi-cycle
$b_{0}\in \left[3.94,5\right]$	chaotic		

**Table 4:** Comparison of SE complexity of this paper with the other literature.

Reference	System Dimension	$SE_{max}$
ours	5	0.65
[32]	5	0.60
[33]	3	0.52
[34]	3	0.53
[35]	4	0.53

**Table 5:** Relevance of the adjacent pixels of the Lena image.

Arithmetic	Horizontal	Vertically	Diagonally
original image	0.98860	0.98197	0.97485
ours	−0.02099	0.01668	0.00132
[38]	0.0635	0.1981	0.1698
[39]	−0.0208	0.0424	0.0212
[40]	−0.0519	−0.0385	0.0046

**Table 6:** Comparison of Lena’s information entropy.

Arithmetic	Channel			Mean
	R	G	B	
ours	7.9992	7.9992	7.9994	7.9993
[41]	7.9997	7.9937	7.9976	7.9970
[42]	7.9991	7.9993	7.9993	7.9992
[43]	7.9914	7.9907	7.9907	7.9909

**Table 7:** The NPCR and UACI results compared with the other referenced literature.

Arithmetic	NPCR	Difference from Ideal Value	UACI	Difference from Ideal Value
ours	99.6103%	0.0009	33.4692%	0.0057
[45]	99.6084%	0.0010	33.4513%	0.0122
[46]	99.6081%	0.0013	33.4478%	0.0157

**Table 8:** Adding varying degrees of salt-and-pepper noise to the PSNR of the decrypted images.

Image	PSNR		
	0.01% Salt-and-Pepper noise	0.1% Salt-and-Pepper Noise	1% Salt-and-Pepper Noise
Lena	33.7643	26.7735	17.1403
Baboon	34.1943	26.8923	17.5167
Peppers	31.5241	25.7498	16.5099

**Table 9:** Adding varying degrees of noise to the PSNR of the decrypted images.

PSNR	Image		
	Lena	Baboon	Peppers
Gaussian noise with variance $1\times 10^{-10}$	37.0769	37.3495	33.3407
Gaussian noise with variance $1\times 10^{-6}$	11.5303	11.8778	10.8550
Gaussian noise with variance $2\times 10^{-6}$	7.49710	7.89210	6.93400

**Table 10:** Decrypting PSNR and SSIM of images.

Image		PSNR	SSIM
encrypted image	Lena	3.8448	0.0075332
	Baboon	4.0104	0.0093269
	Peppers	3.3049	0.0072834
decrypted image	Lena	37.0769	0.99959
	Baboon	37.3495	0.99948
	Peppers	33.3407	0.99934

## Data Availability

The data presented in this study are available on request from the corresponding author.
